# Supplementary material for: Nutrition Education on the Wards: A Self-Study Module for Improving Medical Student Knowledge of Nutrition Assessment and Interventions
Source: MedEdPORTAL. 2020 Oct 16;16:10968. doi: 10.15766/mep_2374-8265.10968 (PMC7566223; doi:10.15766/mep_2374-8265.10968)
Supplement: Supplementary file 1 — Instructions.docxPremodule Nutrition Evaluation Survey.docxNutrition Education Module.pptxPostmodule Nutrition Evaluation Survey.docxAnswer Key.docx [file mep_2374-8265.10968-s001.zip › A. Instructions.docx]

**Appendix A**

**Nutrition Education on the Wards: Instructor’s Guide**

1. Determine the best clerkships to provide the nutrition education module as an independent study tool.
   1. Based on our findings, we recommend this module be administered during a clerkship without a shelf exam such as Critical Care, Subinternship in Medicine or Subinternship in Surgery. If given to third-year medical students during clerkships such as medicine and surgery that have a corresponding clerkship exam, we recommend administering this module as a required assignment that is built into the required didactics early in the clerkship at a time that does not coincide with clerkship exams.

1. Administer the education module (Appendix C) as a pdf file to students via email or other online programs used at your institution.
2. If your institution would like to measure the learning effectiveness of this module, you may use the pre-module nutrition evaluation survey (Appendix B) and the post-module nutrition evaluation survey (Appendix D) and the answer key sheet (Appendix E).
   1. If using the surveys, we recommend using a tool such as Qualtrics to send the surveys out to students via email with a link to the module during the orientation of the clerkship.
   2. Submit the post-module nutrition evaluation survey mid-way through clerkships.
   3. You may change or add questions to the module such as the below questions, all of which have answers noted within the module.
      1. How to estimate calorie and protein needs
      2. Contraindications for enteral nutrition
      3. Indications for parenteral nutrition
