# Supplementary material for: Nutrition Education on the Wards: A Self-Study Module for Improving Medical Student Knowledge of Nutrition Assessment and Interventions
Source: MedEdPORTAL. 2020 Oct 16;16:10968. doi: 10.15766/mep_2374-8265.10968 (PMC7566223; doi:10.15766/mep_2374-8265.10968)
Supplement: Supplementary file 1 — Instructions.docxPremodule Nutrition Evaluation Survey.docxNutrition Education Module.pptxPostmodule Nutrition Evaluation Survey.docxAnswer Key.docx [file mep_2374-8265.10968-s001.zip › B. Premodule Nutrition Evaluation Survey.docx]

**Appendix B**

**Nutrition Education on the Wards: Pre-Module Survey**

1. Which clerkship have you just completed?

- Surgery
- Medicine
- Subinternship in Internal Medicine
- Critical Care

1. Did you have any additional formal nutrition education outside of your M1 and M2 course?

- No
- Yes

1. Have you had any formal nutrition education during your M3 or M4 year?

- Yes during M3
- Yes during M4
- No

1. Have you come across any patient case during your inpatient rotations that required nutrition intervention from a nutrition consult?

- No
- Yes

1. If you came across any patient case during your inpatient rotation that required nutrition intervention, did you understand the nutrition’s team recommendations?

- No
- Yes

1. I have an understanding of basic nutritional requirements such as how to determine general calories needs and macronutrients.

- I do not
- I can state the 3 main macronutrients

1. I understand the role of acute phase serum protein such as albumin in interpreting a patient’s nutritional status

- No
- I know what an acute phase protein is but not how to use it in nutrition assessment.
- Yes

1. I can state at least 2 factors that qualifies a patients to be malnourished

- No
- I can name 1
- Yes

1. I understand the difference between enteral and parenteral nutrition.

- No
- Yes
- I can only describe enteral nutrition
- I can only describe parenteral nutrition

1. I can state 3 reasons why a patient may need enteral feeds

- No
- I can name 1 or 2 reasons
- Yes

1. I can state 3 contraindications for TPN

- No
- I can name 1 or 2 contraindication
- Yes

**Page break - click on arrow to get to the next section if using qualtrics (or student must flip the page)**

1. Please state an acute phase protein and its use in nutrition assessment:
2. State at least 2 factors that qualify a patients to be malnourished:
3. State the difference between enteral and parenteral nutrition:
4. State 3 reasons why a patient may need enteral feeds:
5. State 3 contraindications for TPN:

**Page break - Once the student clicks the next arrow, they will see the bellow message:**

“**Please click next to access the nutrition module.”**

Reminder: This is a voluntary participation and all responses you submit are anonymous. Therefore, once you click next you will not be able to withdraw from participation, as we will not know which response is yours.

**Page break - Once the student clicks next, they will see the below message:**

“Please click on the below link to gain access to the nutrition support education module. 

The module should take no longer than 30 minutes to review. Introduction to Nutrition Education.pdf

“Thank you again for your participation and interest in nutrition support. Your response has been recorded.”
