# Supplementary material for: Nutrition Education on the Wards: A Self-Study Module for Improving Medical Student Knowledge of Nutrition Assessment and Interventions
Source: MedEdPORTAL. 2020 Oct 16;16:10968. doi: 10.15766/mep_2374-8265.10968 (PMC7566223; doi:10.15766/mep_2374-8265.10968)
Supplement: Supplementary file 1 — Instructions.docxPremodule Nutrition Evaluation Survey.docxNutrition Education Module.pptxPostmodule Nutrition Evaluation Survey.docxAnswer Key.docx [file mep_2374-8265.10968-s001.zip › C. Nutrition Education Module.pptx]

## Slide 1
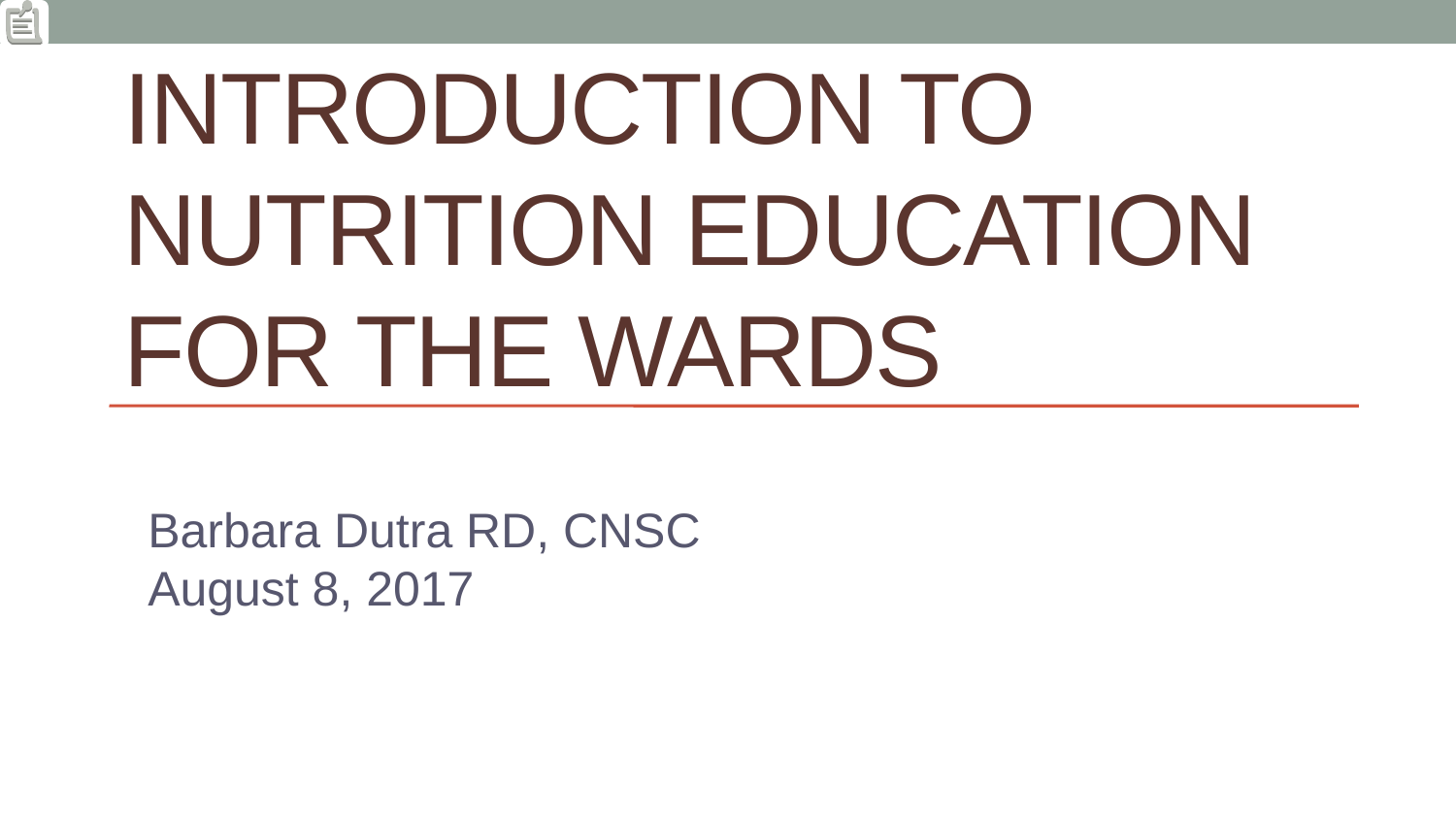

# Introduction to Nutrition Education FOR the Wards
Barbara Dutra RD, CNSC
August 8, 2017

## Slide 2
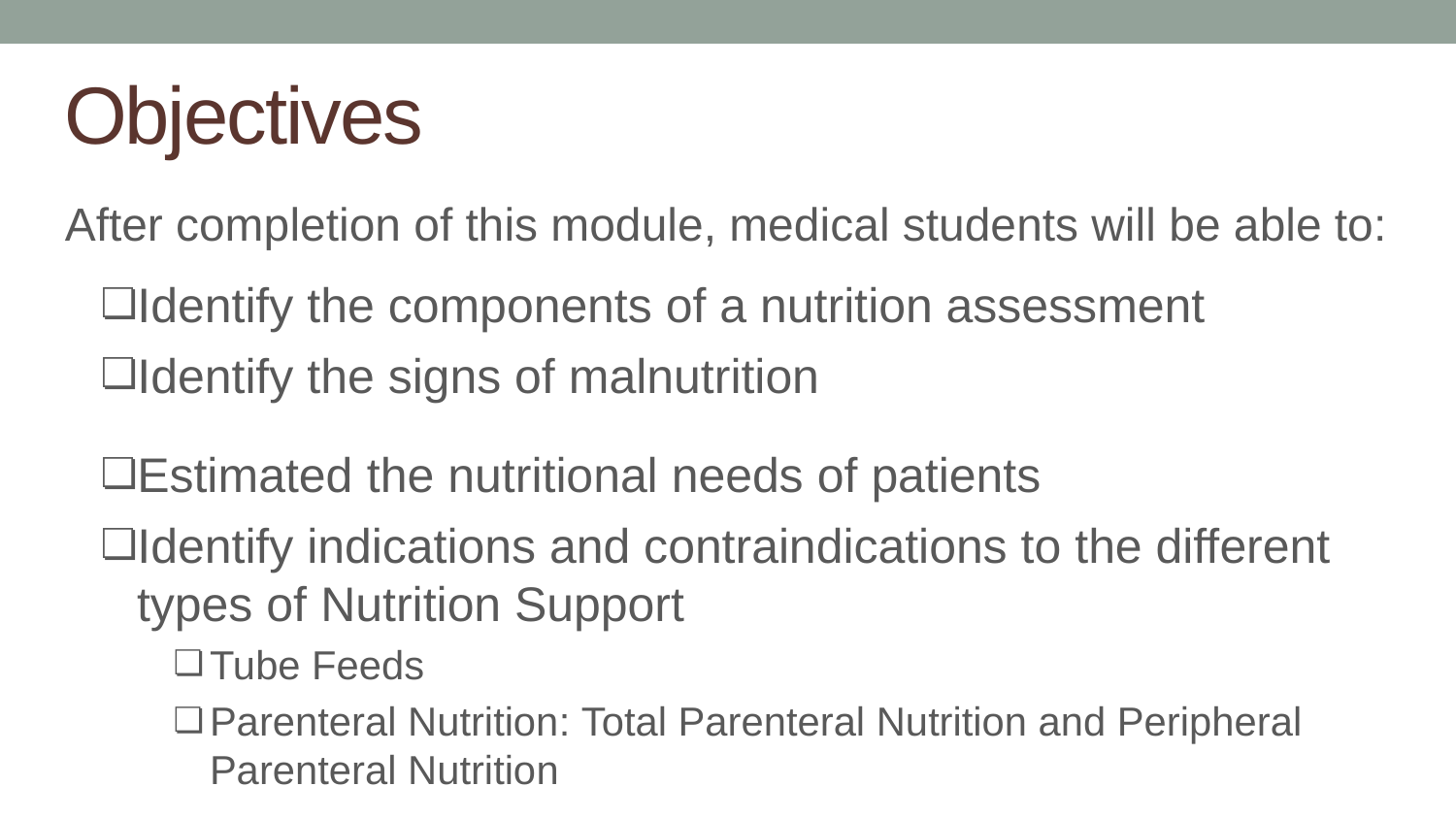

# Objectives
After completion of this module, medical students will be able to:
Identify the components of a nutrition assessment
Identify the signs of malnutrition
Estimated the nutritional needs of patients
Identify indications and contraindications to the different types of Nutrition Support
Tube Feeds
Parenteral Nutrition: Total Parenteral Nutrition and Peripheral Parenteral Nutrition

## Slide 3
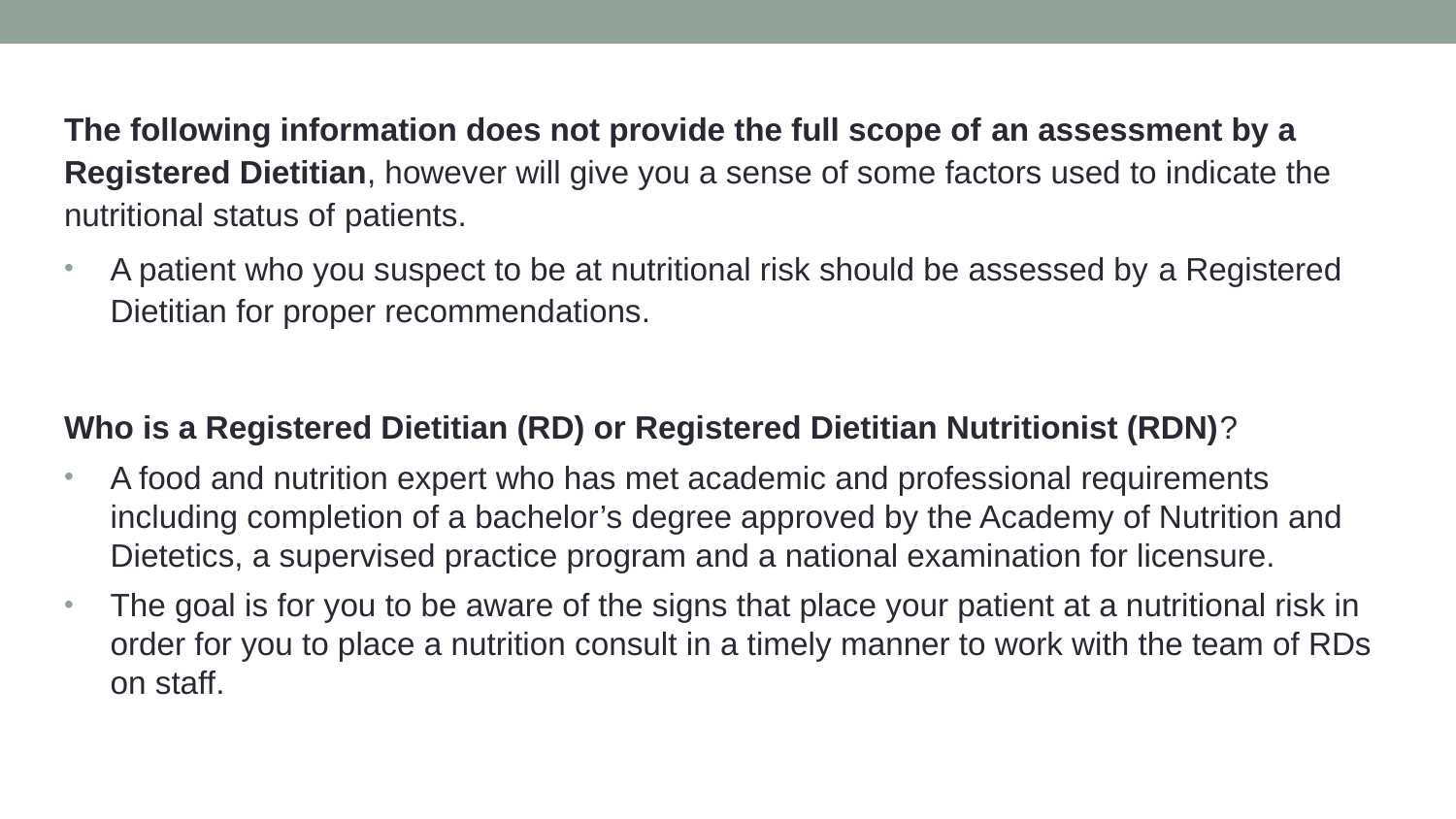

The following information does not provide the full scope of an assessment by a Registered Dietitian, however will give you a sense of some factors used to indicate the nutritional status of patients.
A patient who you suspect to be at nutritional risk should be assessed by a Registered Dietitian for proper recommendations.
Who is a Registered Dietitian (RD) or Registered Dietitian Nutritionist (RDN)?
A food and nutrition expert who has met academic and professional requirements including completion of a bachelor’s degree approved by the Academy of Nutrition and Dietetics, a supervised practice program and a national examination for licensure.
The goal is for you to be aware of the signs that place your patient at a nutritional risk in order for you to place a nutrition consult in a timely manner to work with the team of RDs on staff.

## Slide 4
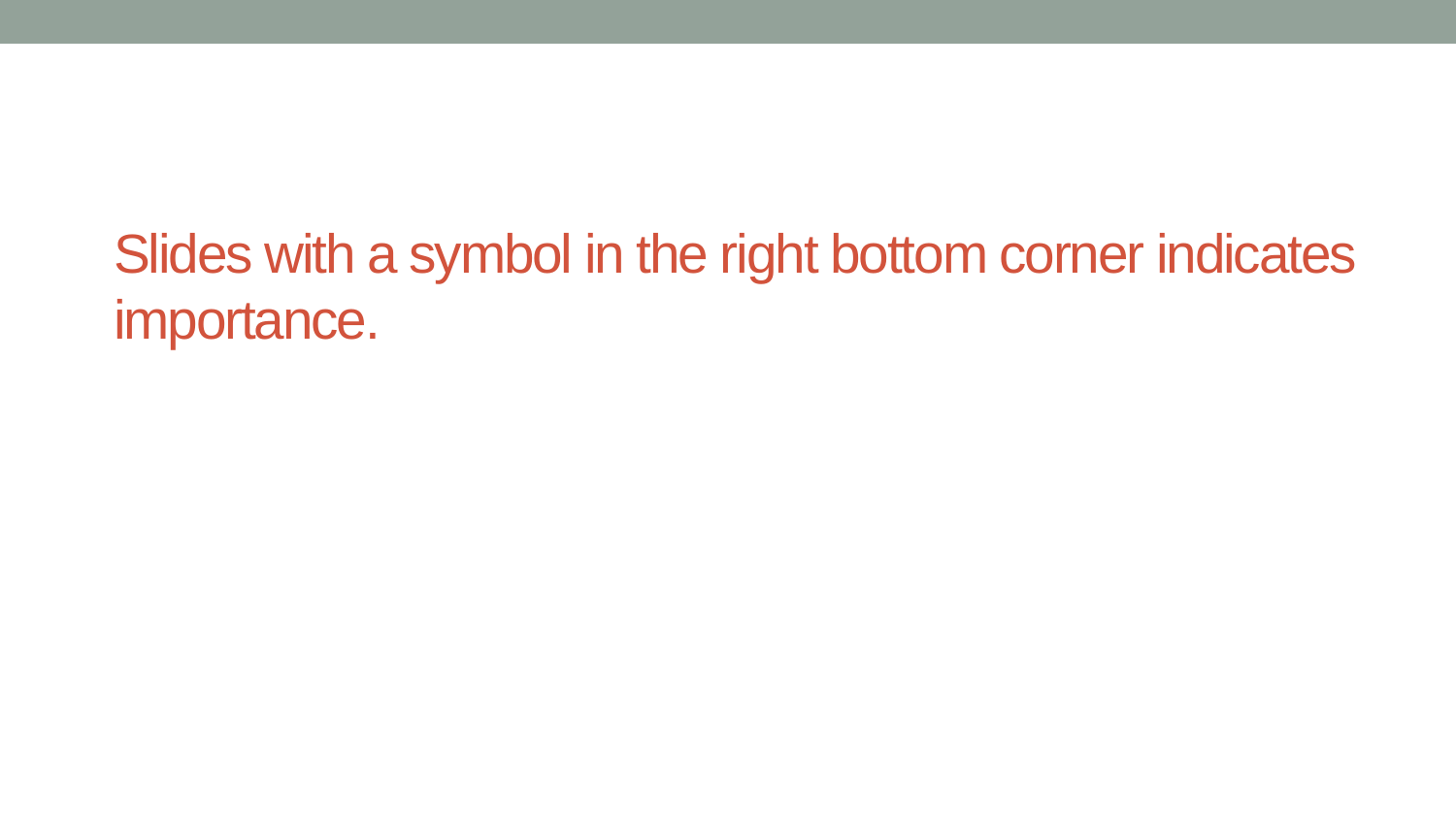

# Slides with a symbol in the right bottom corner indicates importance.

## Slide 5
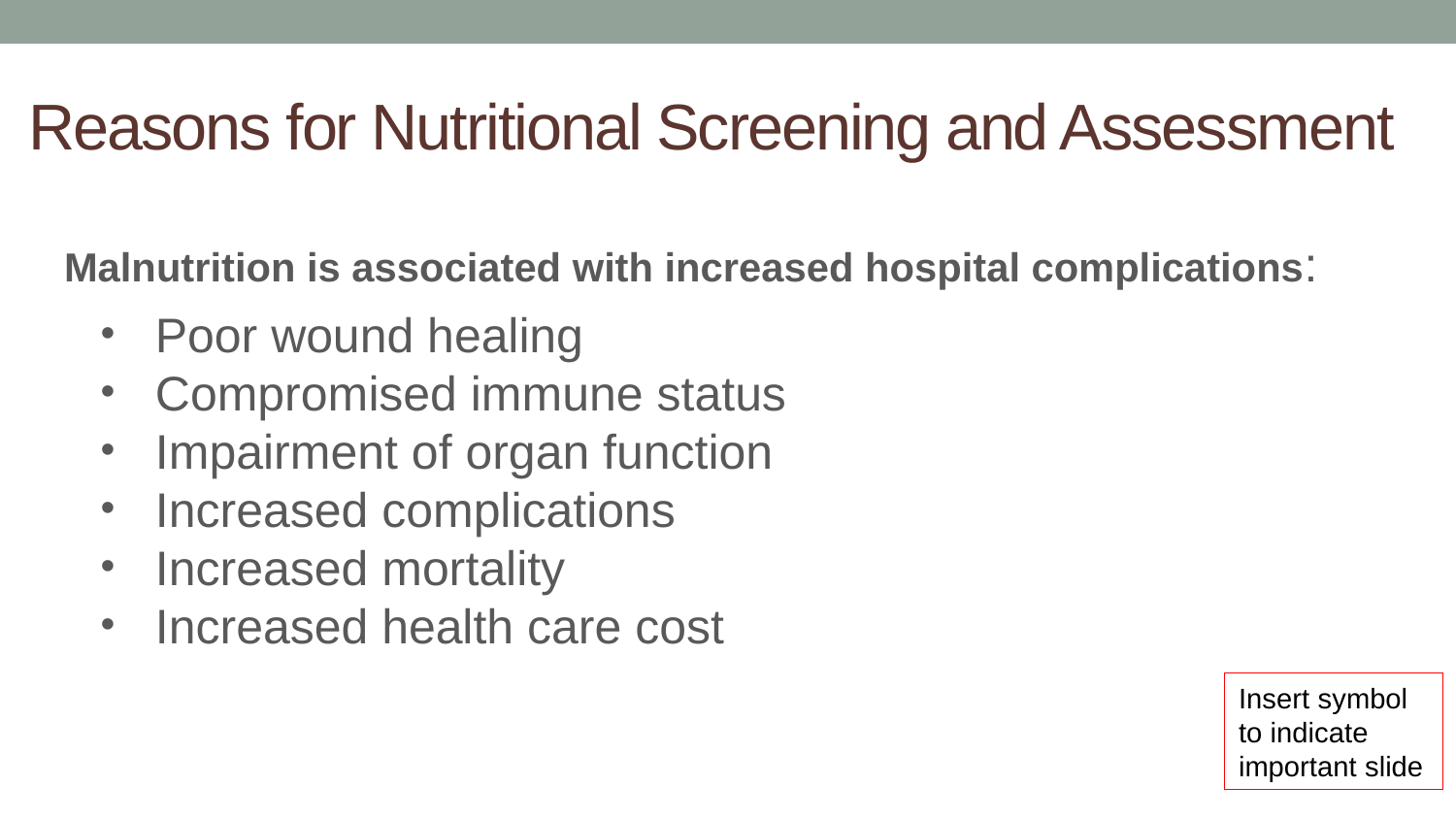

# Reasons for Nutritional Screening and Assessment
Malnutrition is associated with increased hospital complications:
Poor wound healing
Compromised immune status
Impairment of organ function
Increased complications
Increased mortality
Increased health care cost
Insert symbol to indicate important slide

## Slide 6
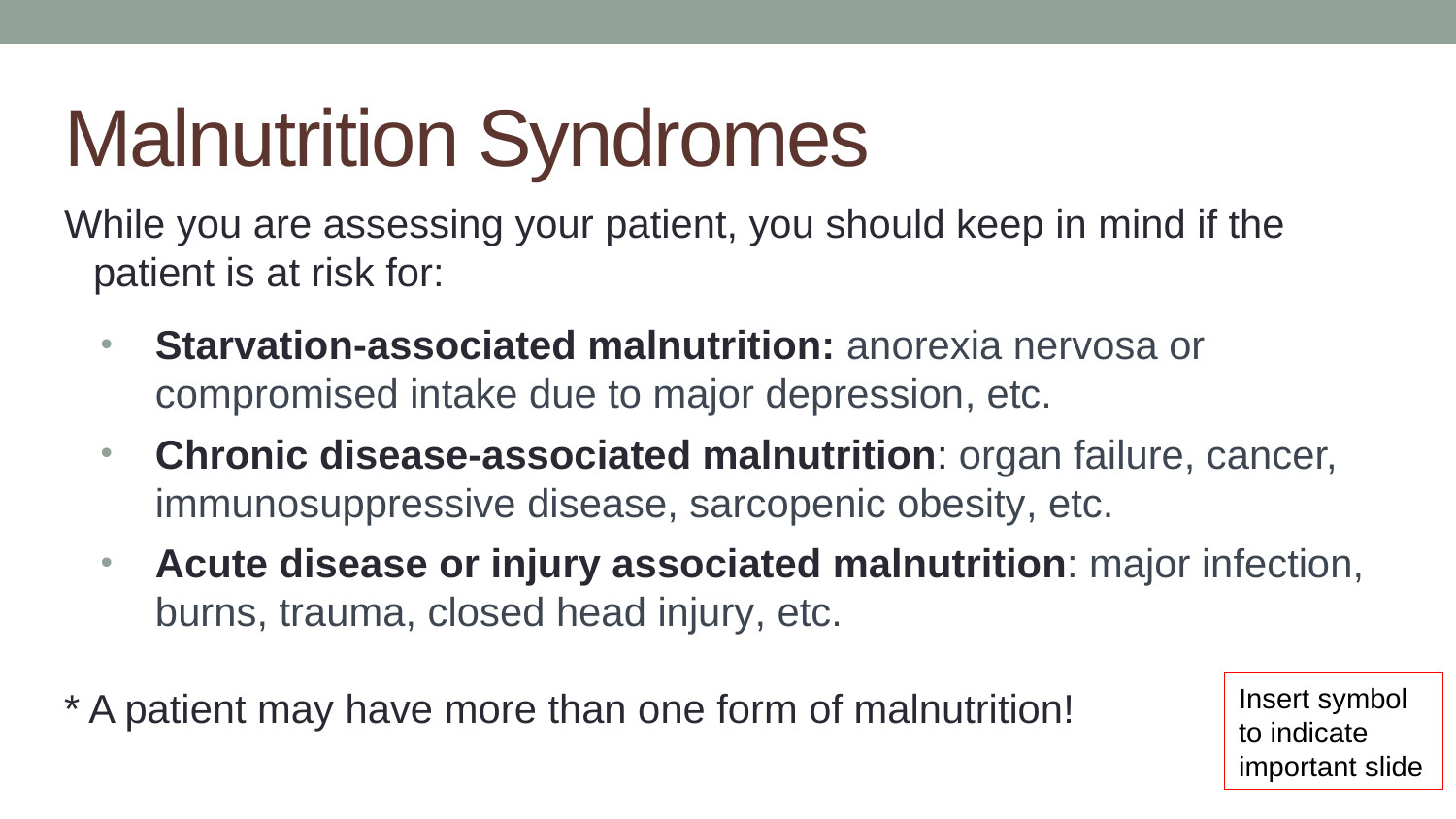

# Malnutrition Syndromes
While you are assessing your patient, you should keep in mind if the patient is at risk for:
Starvation-associated malnutrition: anorexia nervosa or compromised intake due to major depression, etc.
Chronic disease-associated malnutrition: organ failure, cancer, immunosuppressive disease, sarcopenic obesity, etc.
Acute disease or injury associated malnutrition: major infection, burns, trauma, closed head injury, etc.
* A patient may have more than one form of malnutrition!
Insert symbol to indicate important slide

## Slide 7
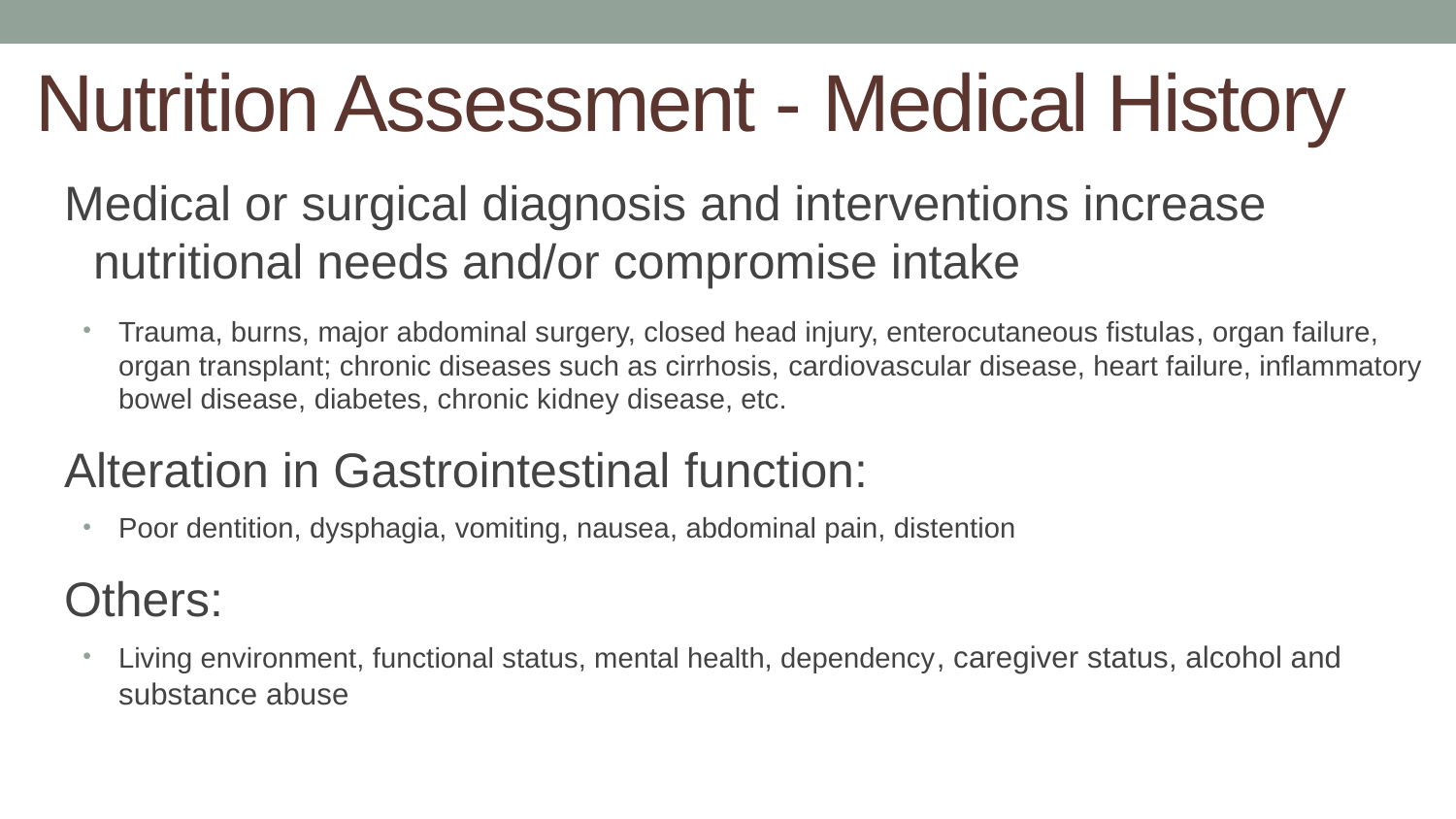

# Nutrition Assessment - Medical History
Medical or surgical diagnosis and interventions increase nutritional needs and/or compromise intake
Trauma, burns, major abdominal surgery, closed head injury, enterocutaneous fistulas, organ failure, organ transplant; chronic diseases such as cirrhosis, cardiovascular disease, heart failure, inflammatory bowel disease, diabetes, chronic kidney disease, etc.
Alteration in Gastrointestinal function:
Poor dentition, dysphagia, vomiting, nausea, abdominal pain, distention
Others:
Living environment, functional status, mental health, dependency, caregiver status, alcohol and substance abuse

## Slide 8
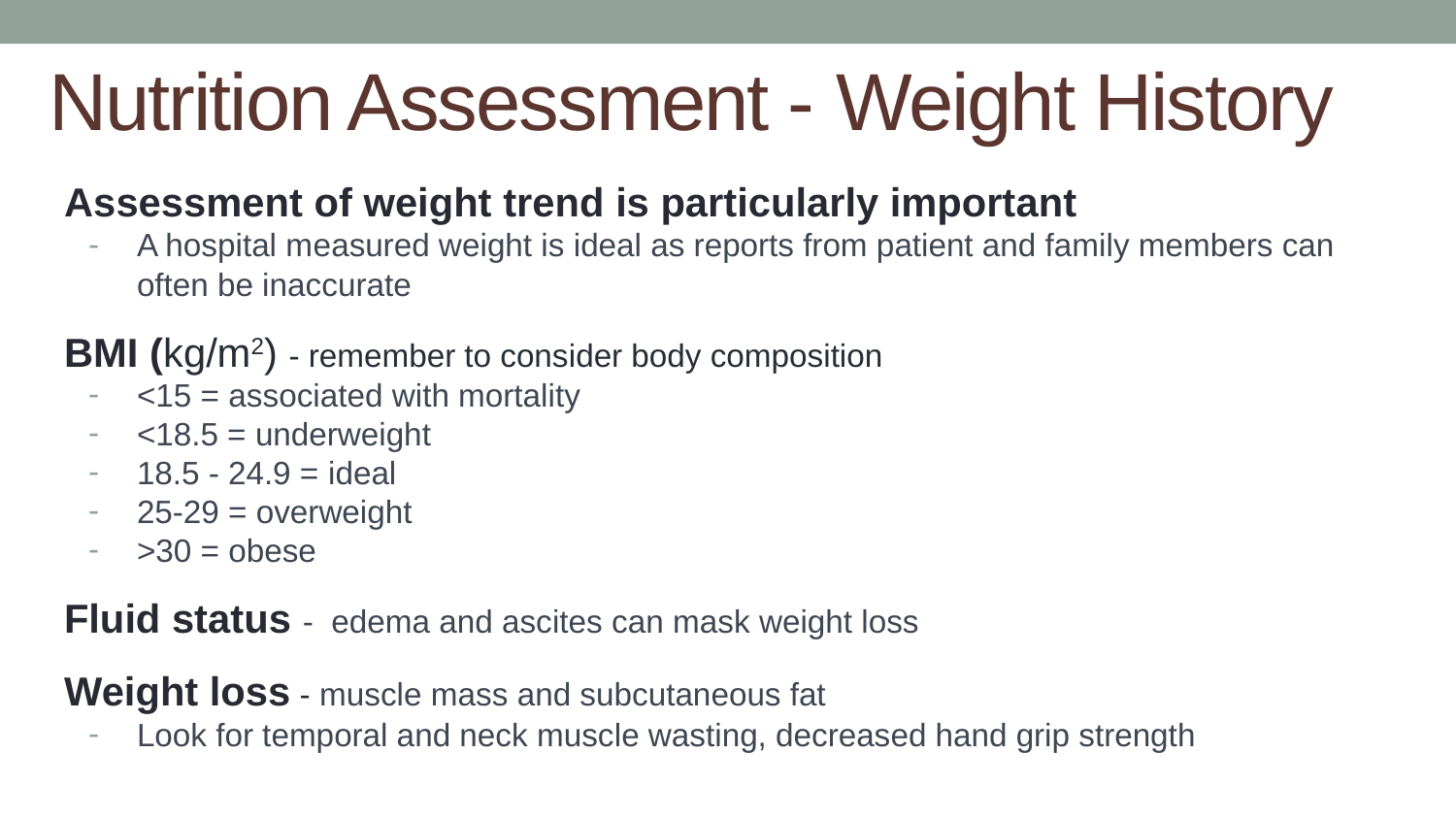

# Nutrition Assessment - Weight History
Assessment of weight trend is particularly important
A hospital measured weight is ideal as reports from patient and family members can often be inaccurate
BMI (kg/m2) - remember to consider body composition
<15 = associated with mortality
<18.5 = underweight
18.5 - 24.9 = ideal
25-29 = overweight
>30 = obese
Fluid status - edema and ascites can mask weight loss
Weight loss - muscle mass and subcutaneous fat
Look for temporal and neck muscle wasting, decreased hand grip strength

## Slide 9
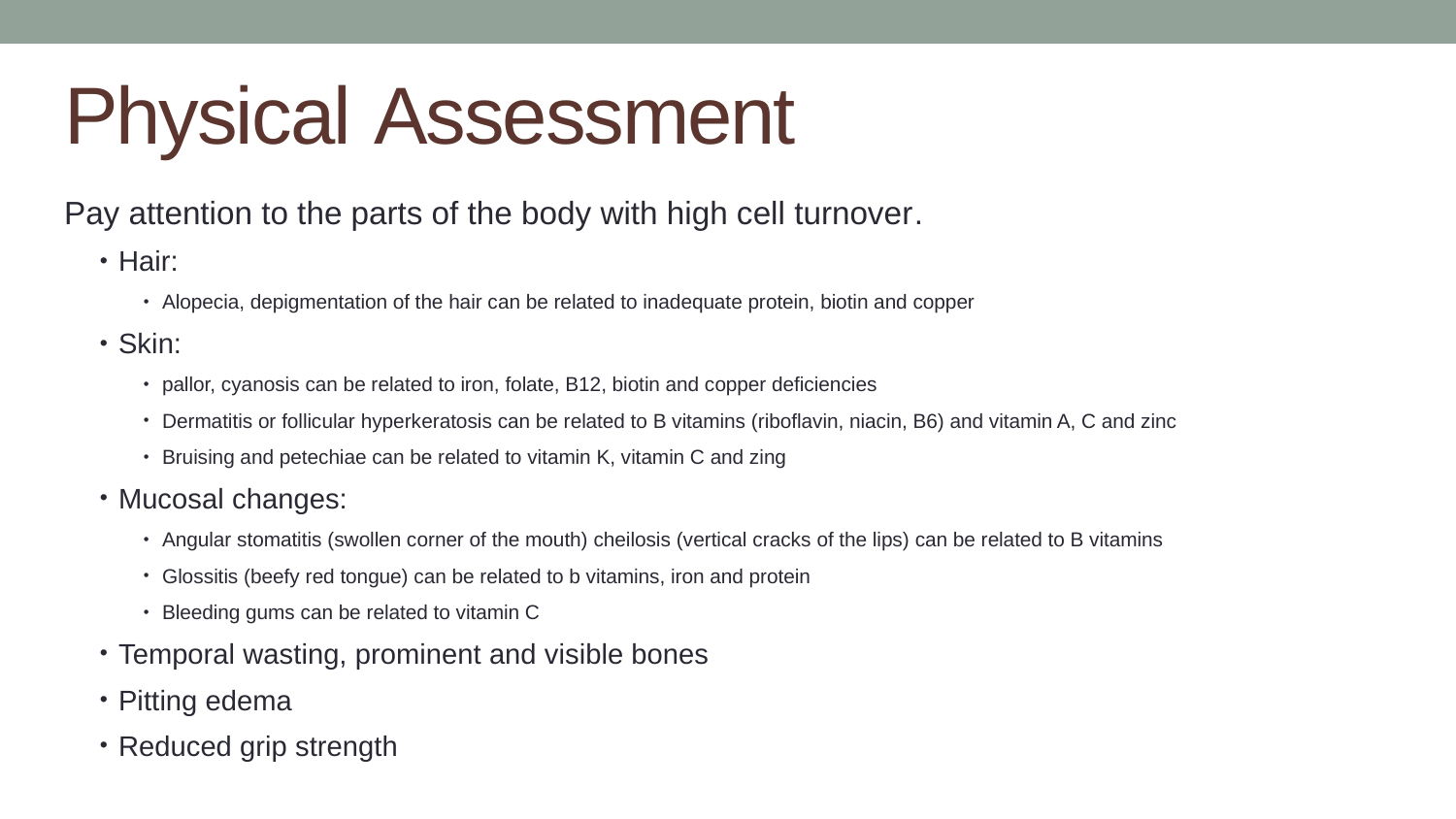

# Physical Assessment
Pay attention to the parts of the body with high cell turnover.
Hair:
Alopecia, depigmentation of the hair can be related to inadequate protein, biotin and copper
Skin:
pallor, cyanosis can be related to iron, folate, B12, biotin and copper deficiencies
Dermatitis or follicular hyperkeratosis can be related to B vitamins (riboflavin, niacin, B6) and vitamin A, C and zinc
Bruising and petechiae can be related to vitamin K, vitamin C and zing
Mucosal changes:
Angular stomatitis (swollen corner of the mouth) cheilosis (vertical cracks of the lips) can be related to B vitamins
Glossitis (beefy red tongue) can be related to b vitamins, iron and protein
Bleeding gums can be related to vitamin C
Temporal wasting, prominent and visible bones
Pitting edema
Reduced grip strength

## Slide 10
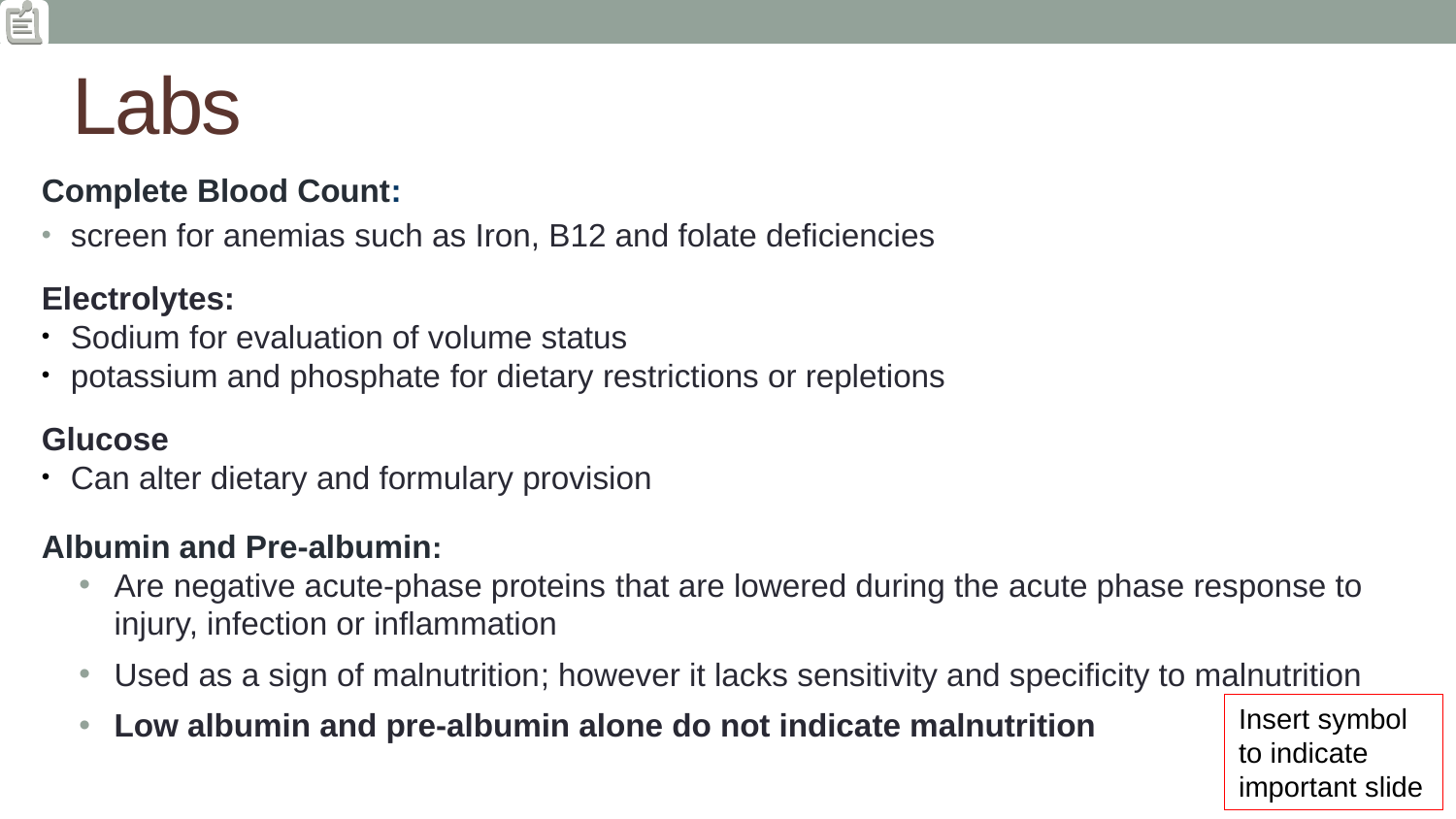

# Labs
Complete Blood Count:
screen for anemias such as Iron, B12 and folate deficiencies
Electrolytes:
Sodium for evaluation of volume status
potassium and phosphate for dietary restrictions or repletions
Glucose
Can alter dietary and formulary provision
Albumin and Pre-albumin:
Are negative acute-phase proteins that are lowered during the acute phase response to injury, infection or inflammation
Used as a sign of malnutrition; however it lacks sensitivity and specificity to malnutrition
Low albumin and pre-albumin alone do not indicate malnutrition
Insert symbol to indicate important slide

## Slide 11
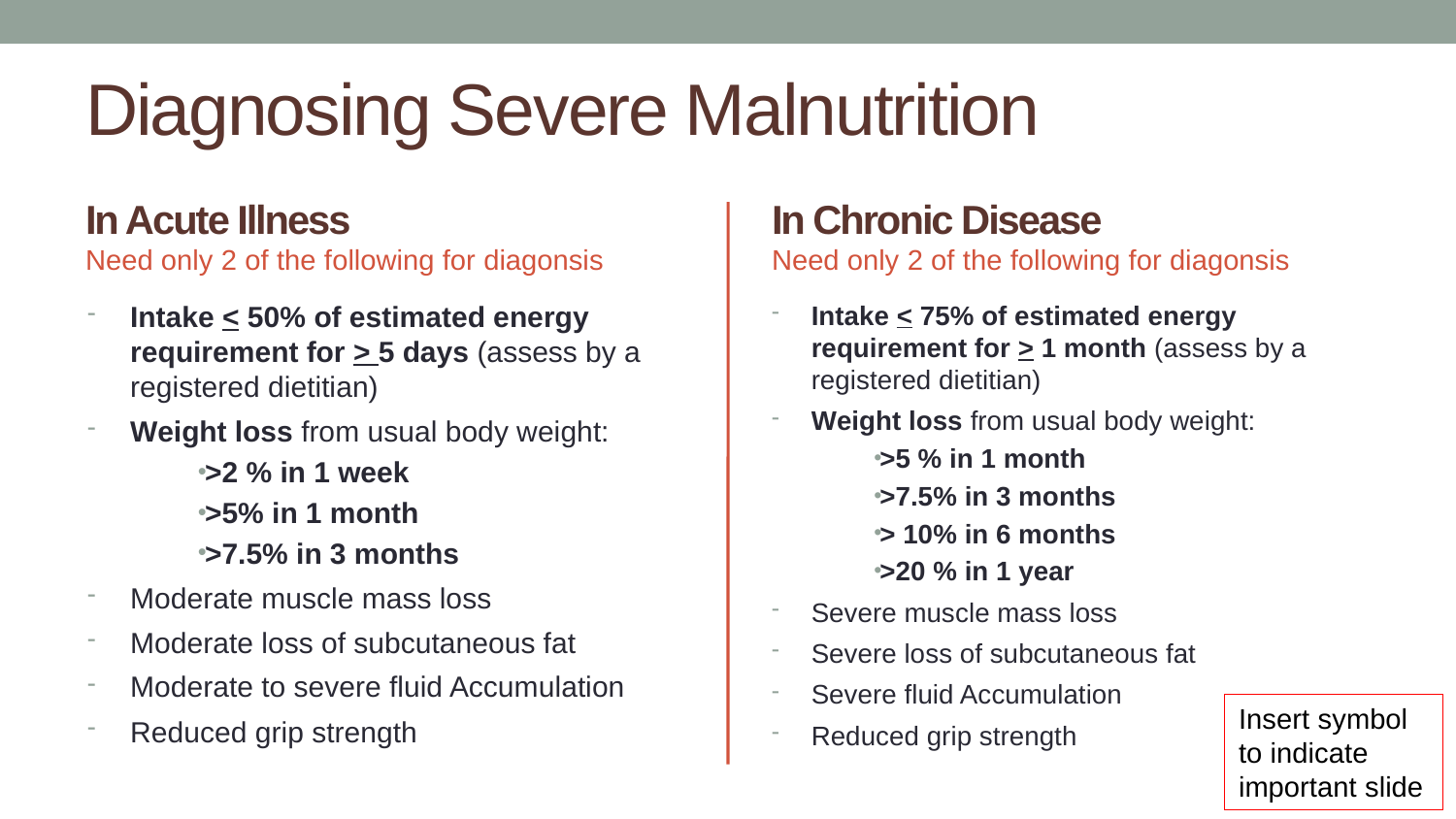

# Diagnosing Severe Malnutrition
In Acute Illness
Need only 2 of the following for diagonsis
In Chronic Disease
Need only 2 of the following for diagonsis
Intake < 50% of estimated energy requirement for > 5 days (assess by a registered dietitian)
Weight loss from usual body weight:
>2 % in 1 week
>5% in 1 month
>7.5% in 3 months
Moderate muscle mass loss
Moderate loss of subcutaneous fat
Moderate to severe fluid Accumulation
Reduced grip strength
Intake < 75% of estimated energy requirement for > 1 month (assess by a registered dietitian)
Weight loss from usual body weight:
>5 % in 1 month
>7.5% in 3 months
> 10% in 6 months
>20 % in 1 year
Severe muscle mass loss
Severe loss of subcutaneous fat
Severe fluid Accumulation
Reduced grip strength
Insert symbol to indicate important slide

## Slide 12
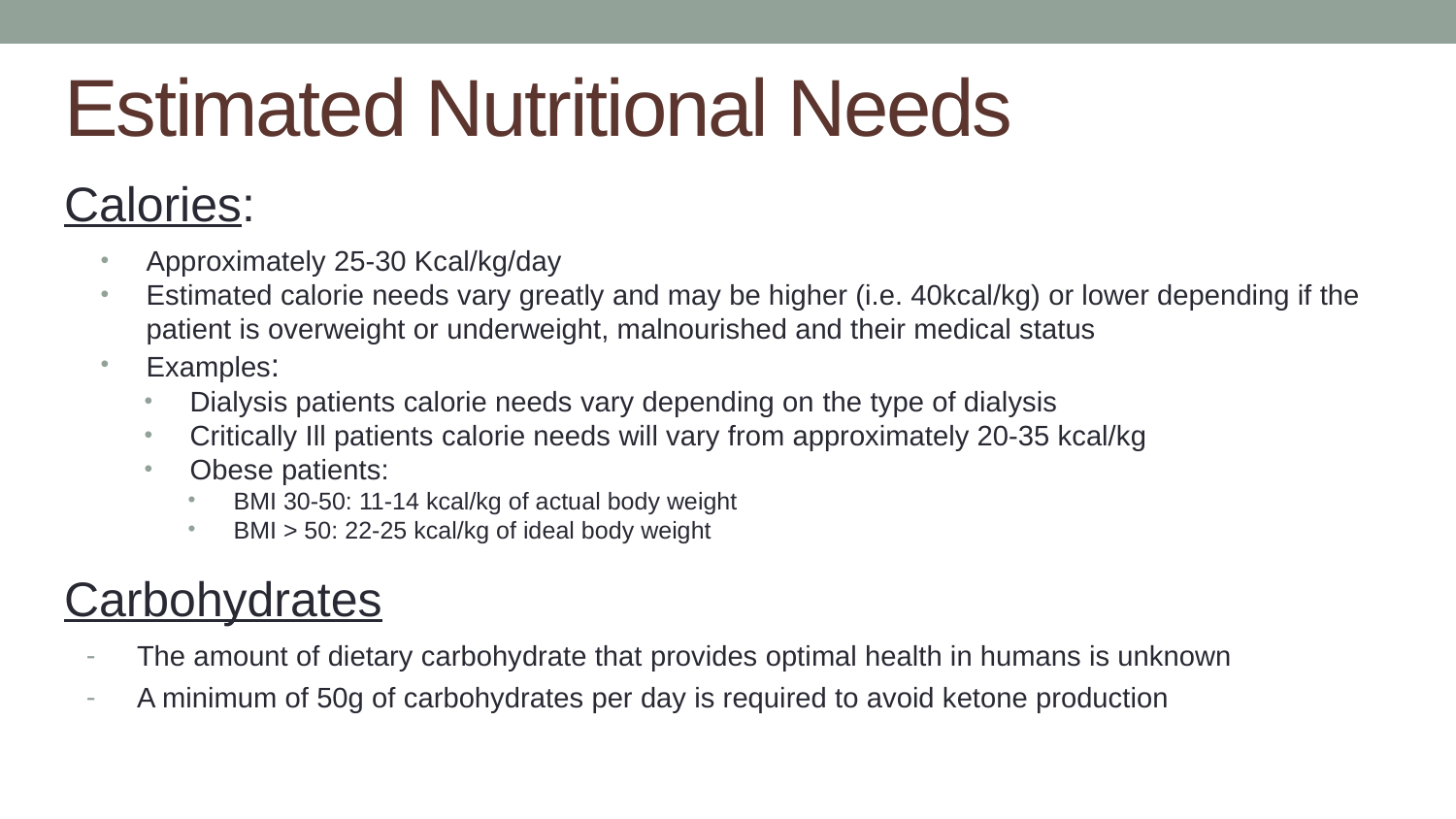

# Estimated Nutritional Needs
Calories:
Approximately 25-30 Kcal/kg/day
Estimated calorie needs vary greatly and may be higher (i.e. 40kcal/kg) or lower depending if the patient is overweight or underweight, malnourished and their medical status
Examples:
Dialysis patients calorie needs vary depending on the type of dialysis
Critically Ill patients calorie needs will vary from approximately 20-35 kcal/kg
Obese patients:
BMI 30-50: 11-14 kcal/kg of actual body weight
BMI > 50: 22-25 kcal/kg of ideal body weight
Carbohydrates
The amount of dietary carbohydrate that provides optimal health in humans is unknown
A minimum of 50g of carbohydrates per day is required to avoid ketone production

## Slide 13
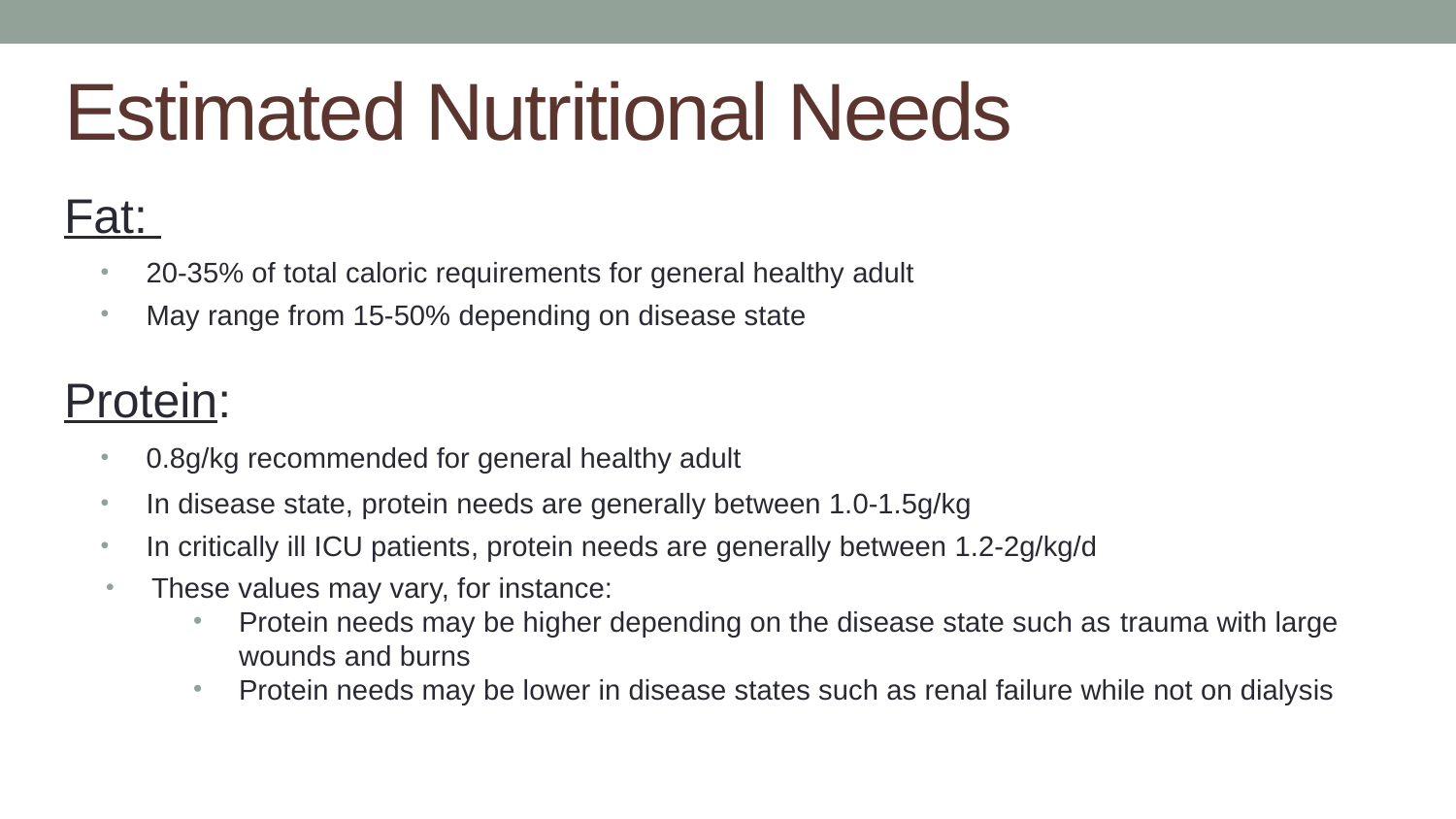

# Estimated Nutritional Needs
Fat:
20-35% of total caloric requirements for general healthy adult
May range from 15-50% depending on disease state
Protein:
0.8g/kg recommended for general healthy adult
In disease state, protein needs are generally between 1.0-1.5g/kg
In critically ill ICU patients, protein needs are generally between 1.2-2g/kg/d
These values may vary, for instance:
Protein needs may be higher depending on the disease state such as trauma with large wounds and burns
Protein needs may be lower in disease states such as renal failure while not on dialysis

## Slide 14
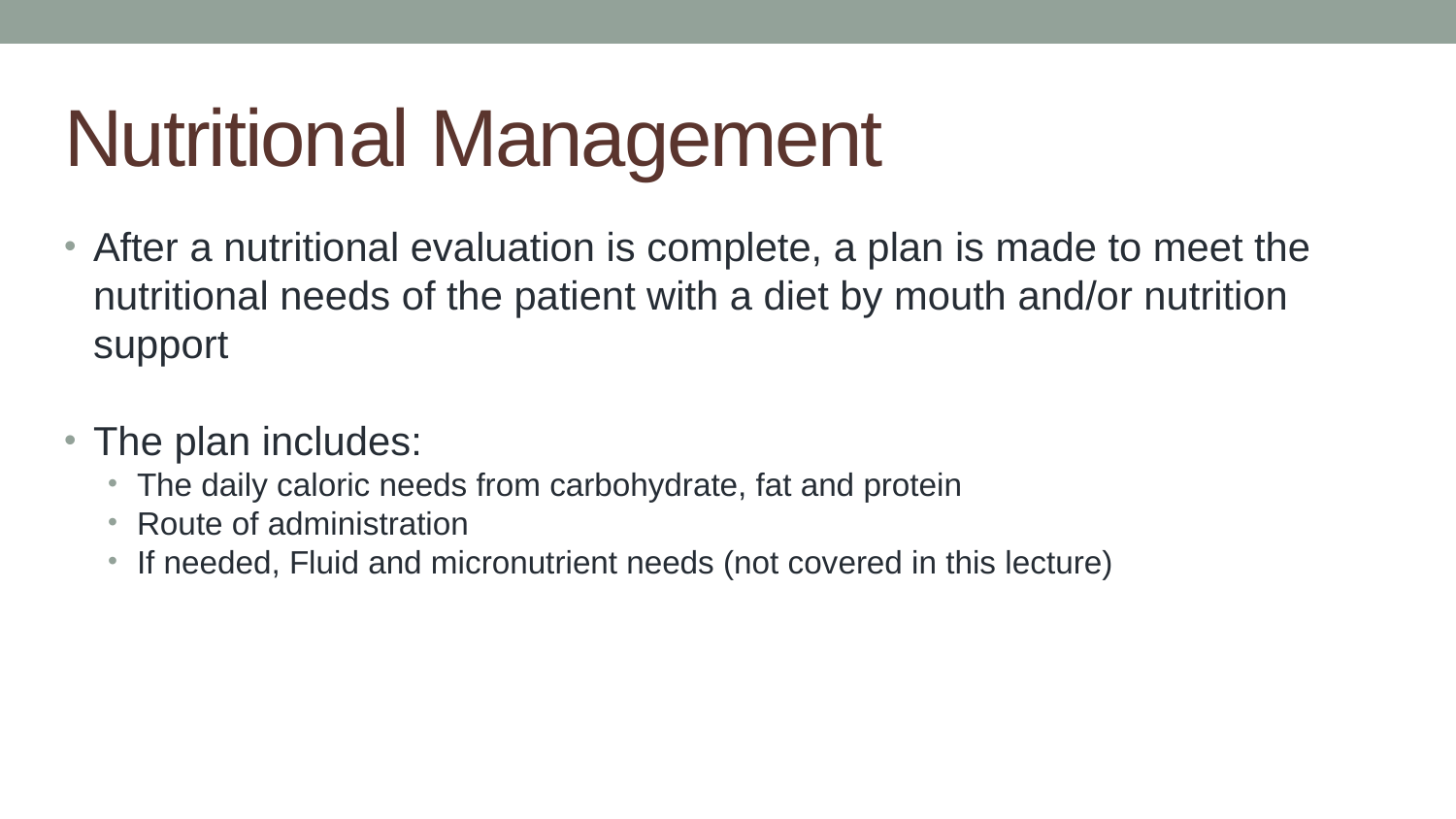

# Nutritional Management
After a nutritional evaluation is complete, a plan is made to meet the nutritional needs of the patient with a diet by mouth and/or nutrition support
The plan includes:
The daily caloric needs from carbohydrate, fat and protein
Route of administration
If needed, Fluid and micronutrient needs (not covered in this lecture)

## Slide 15
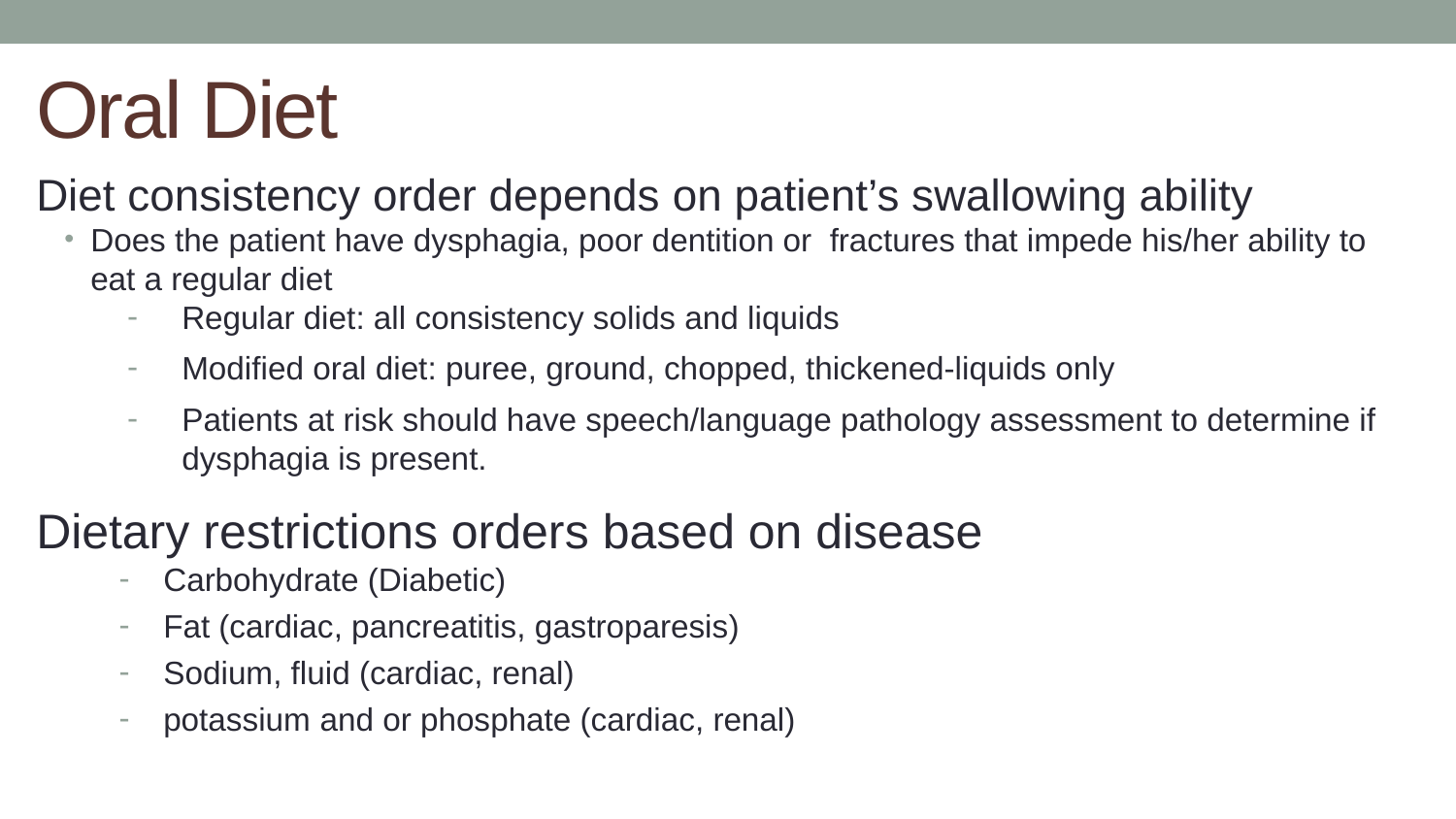

# Oral Diet
Diet consistency order depends on patient’s swallowing ability
Does the patient have dysphagia, poor dentition or fractures that impede his/her ability to eat a regular diet
Regular diet: all consistency solids and liquids
Modified oral diet: puree, ground, chopped, thickened-liquids only
Patients at risk should have speech/language pathology assessment to determine if dysphagia is present.
Dietary restrictions orders based on disease
Carbohydrate (Diabetic)
Fat (cardiac, pancreatitis, gastroparesis)
Sodium, fluid (cardiac, renal)
potassium and or phosphate (cardiac, renal)

## Slide 16
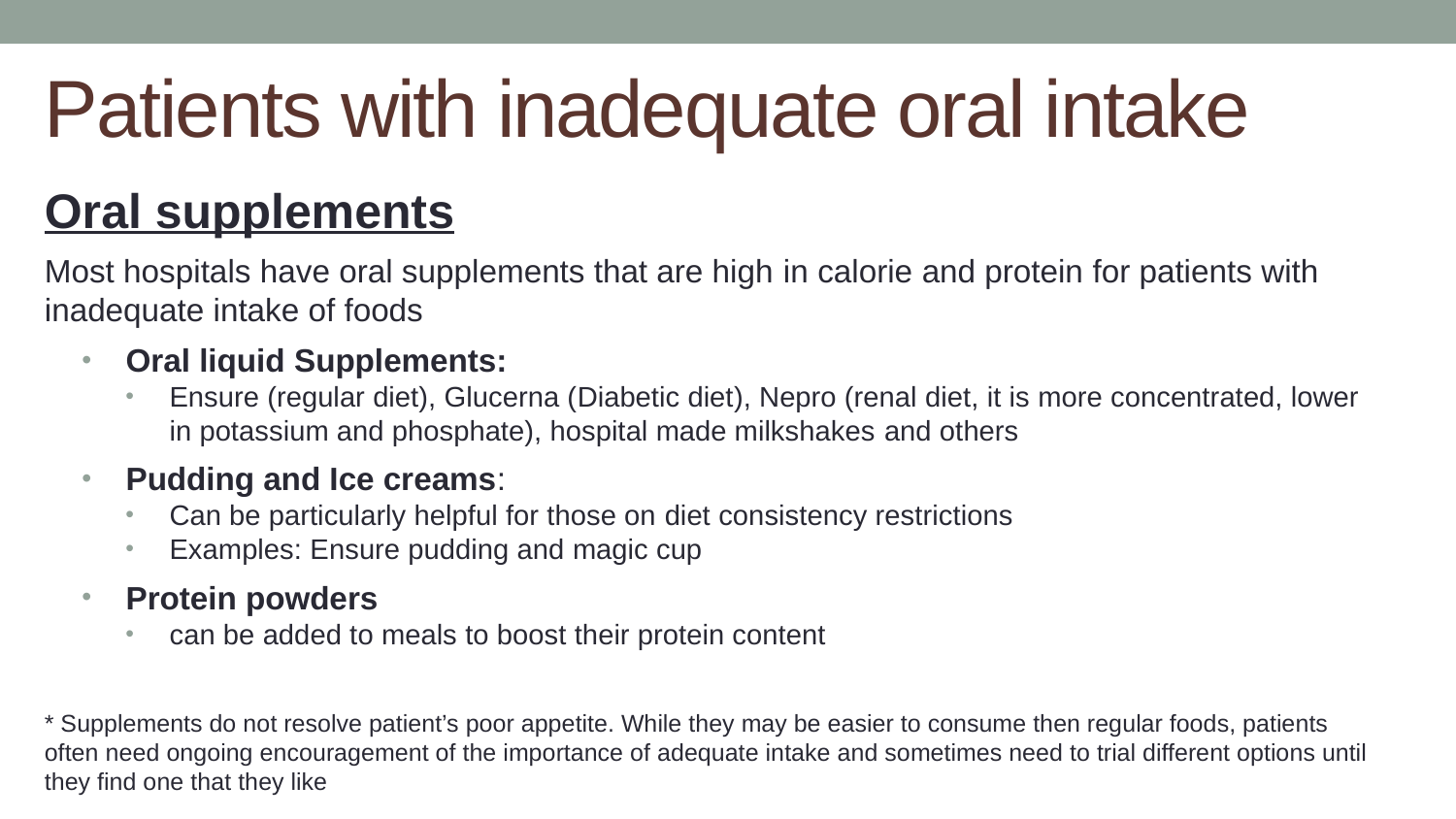

# Patients with inadequate oral intake
Oral supplements
Most hospitals have oral supplements that are high in calorie and protein for patients with inadequate intake of foods
Oral liquid Supplements:
Ensure (regular diet), Glucerna (Diabetic diet), Nepro (renal diet, it is more concentrated, lower in potassium and phosphate), hospital made milkshakes and others
Pudding and Ice creams:
Can be particularly helpful for those on diet consistency restrictions
Examples: Ensure pudding and magic cup
Protein powders
can be added to meals to boost their protein content
* Supplements do not resolve patient’s poor appetite. While they may be easier to consume then regular foods, patients often need ongoing encouragement of the importance of adequate intake and sometimes need to trial different options until they find one that they like

## Slide 17
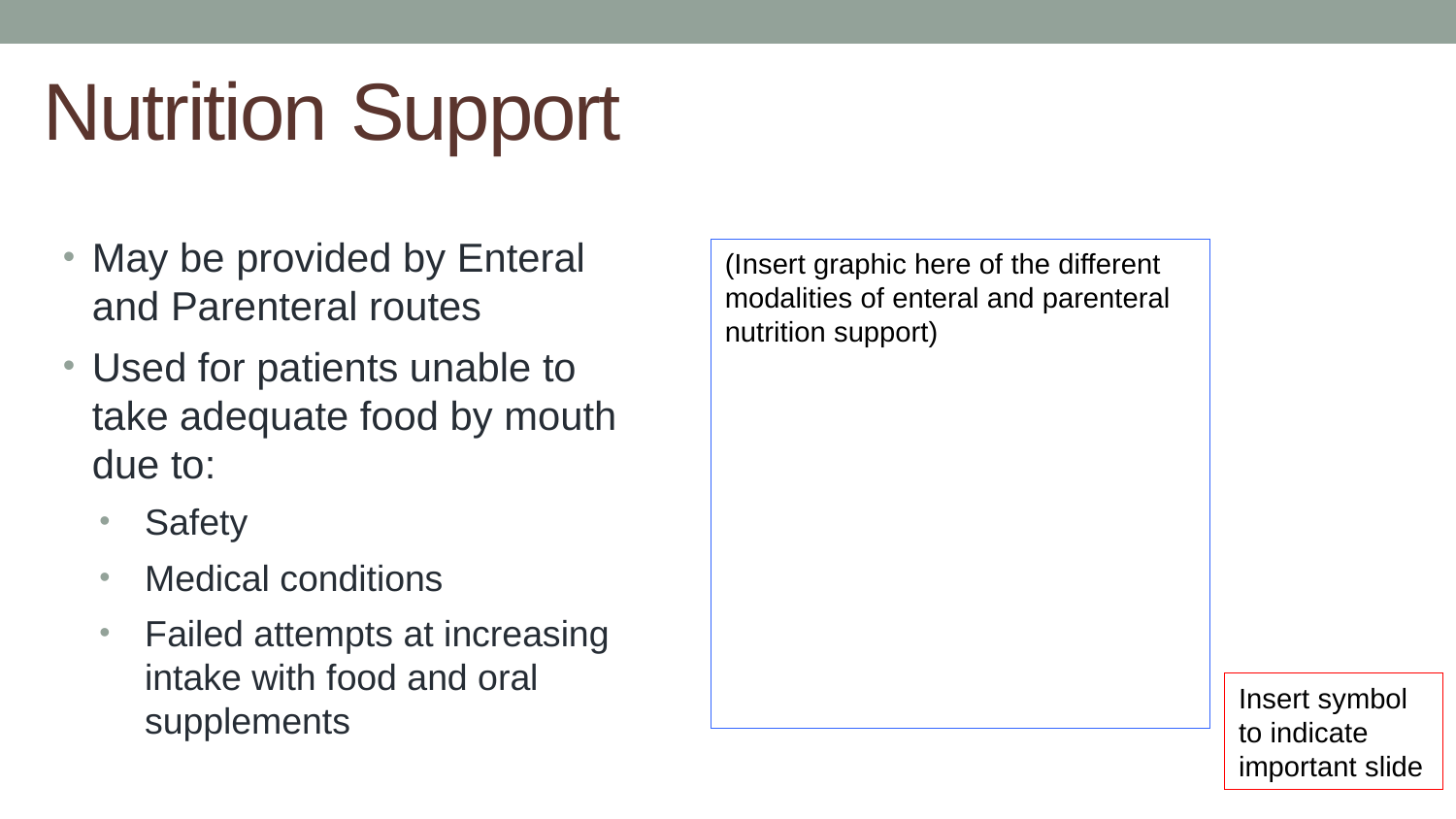

# Nutrition Support
May be provided by Enteral and Parenteral routes
Used for patients unable to take adequate food by mouth due to:
Safety
Medical conditions
Failed attempts at increasing intake with food and oral supplements
(Insert graphic here of the different modalities of enteral and parenteral nutrition support)
Insert symbol to indicate important slide

## Slide 18
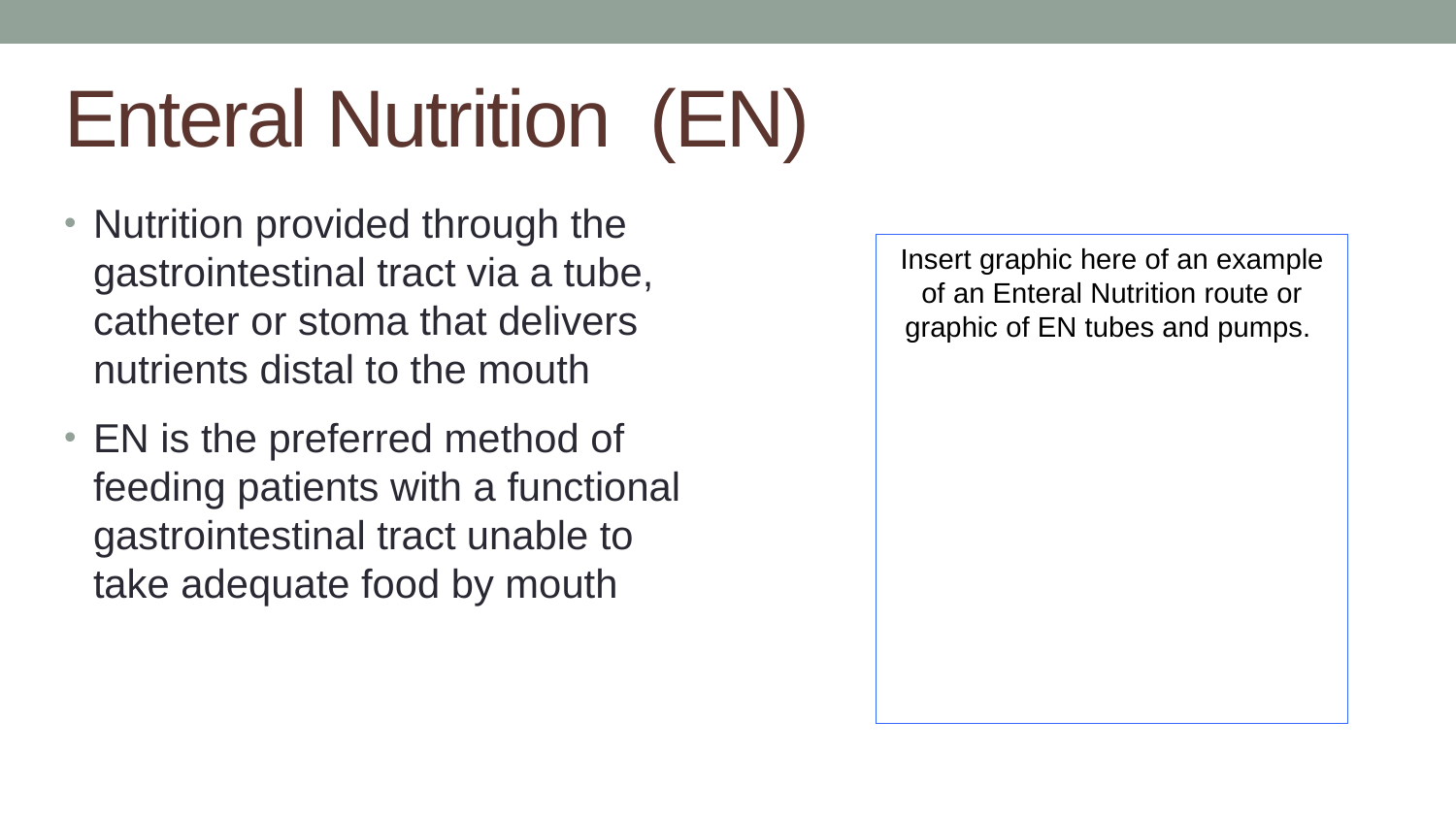

# Enteral Nutrition (EN)
Nutrition provided through the gastrointestinal tract via a tube, catheter or stoma that delivers nutrients distal to the mouth
EN is the preferred method of feeding patients with a functional gastrointestinal tract unable to take adequate food by mouth
Insert graphic here of an example of an Enteral Nutrition route or graphic of EN tubes and pumps.

## Slide 19
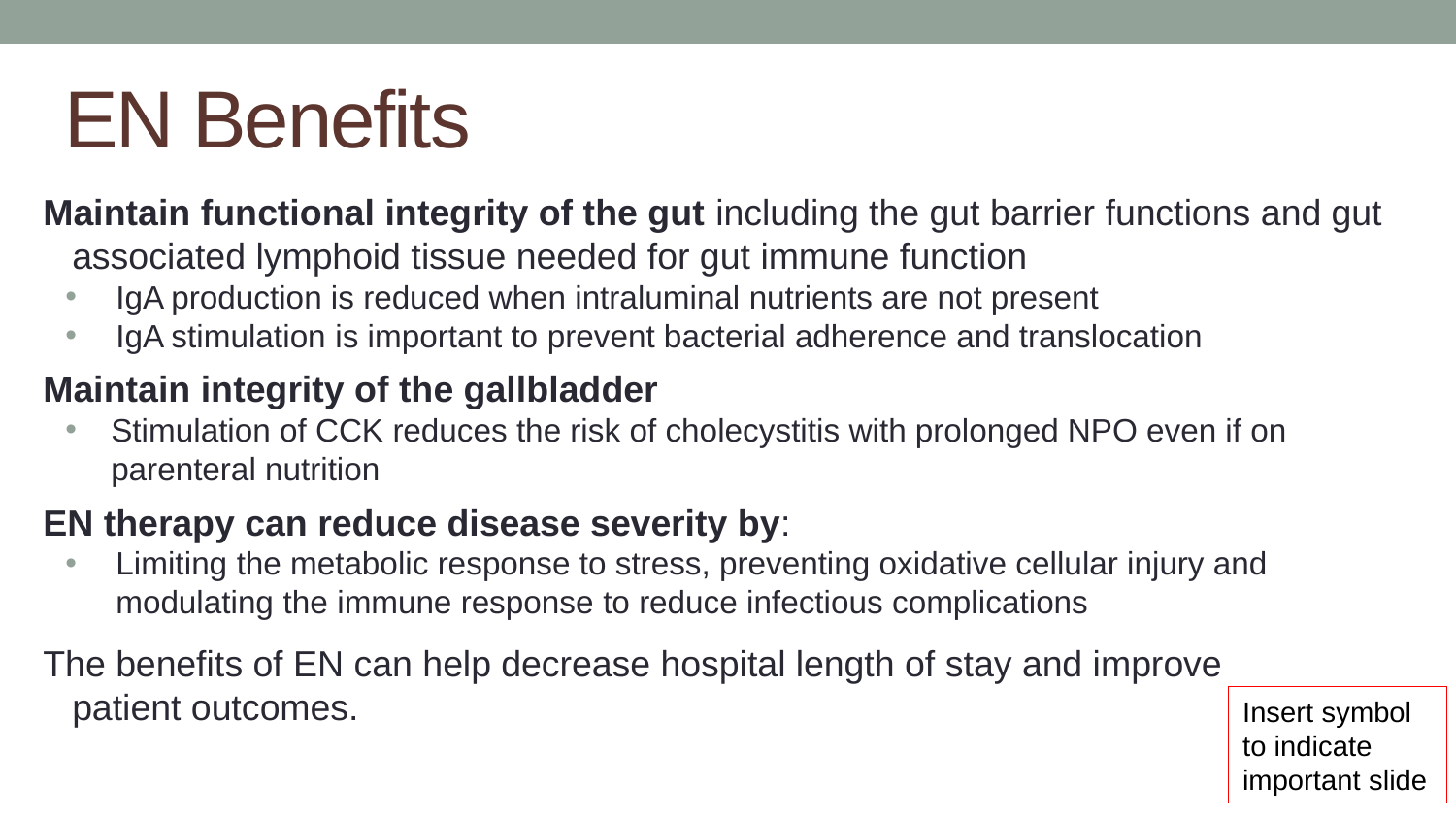

# EN Benefits
Maintain functional integrity of the gut including the gut barrier functions and gut associated lymphoid tissue needed for gut immune function
IgA production is reduced when intraluminal nutrients are not present
IgA stimulation is important to prevent bacterial adherence and translocation
Maintain integrity of the gallbladder
Stimulation of CCK reduces the risk of cholecystitis with prolonged NPO even if on parenteral nutrition
EN therapy can reduce disease severity by:
Limiting the metabolic response to stress, preventing oxidative cellular injury and modulating the immune response to reduce infectious complications
The benefits of EN can help decrease hospital length of stay and improve patient outcomes.
Insert symbol to indicate important slide

## Slide 20
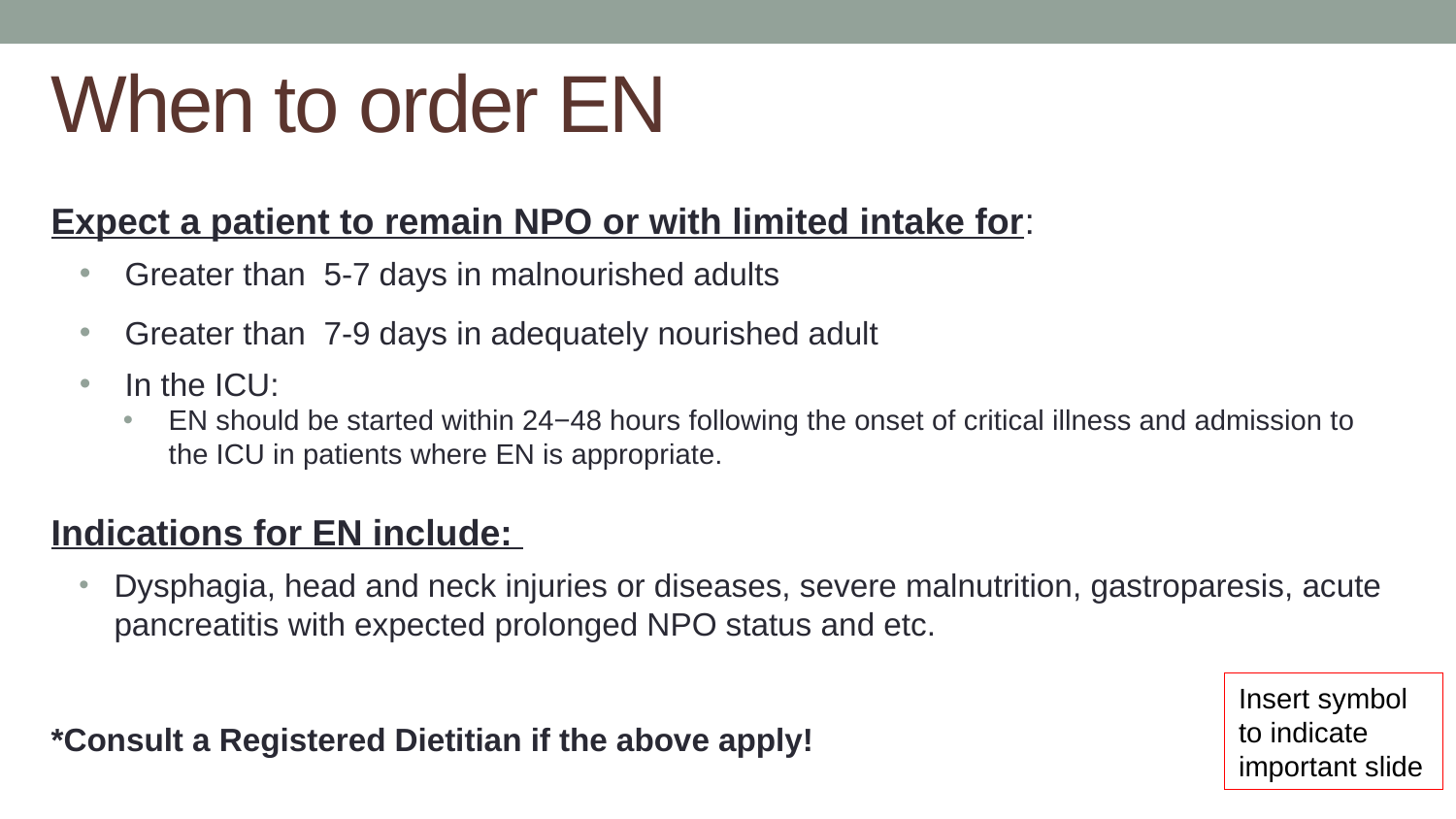

# When to order EN
Expect a patient to remain NPO or with limited intake for:
Greater than 5-7 days in malnourished adults
Greater than 7-9 days in adequately nourished adult
In the ICU:
EN should be started within 24−48 hours following the onset of critical illness and admission to the ICU in patients where EN is appropriate.
Indications for EN include:
Dysphagia, head and neck injuries or diseases, severe malnutrition, gastroparesis, acute pancreatitis with expected prolonged NPO status and etc.
*Consult a Registered Dietitian if the above apply!
Insert symbol to indicate important slide

## Slide 21
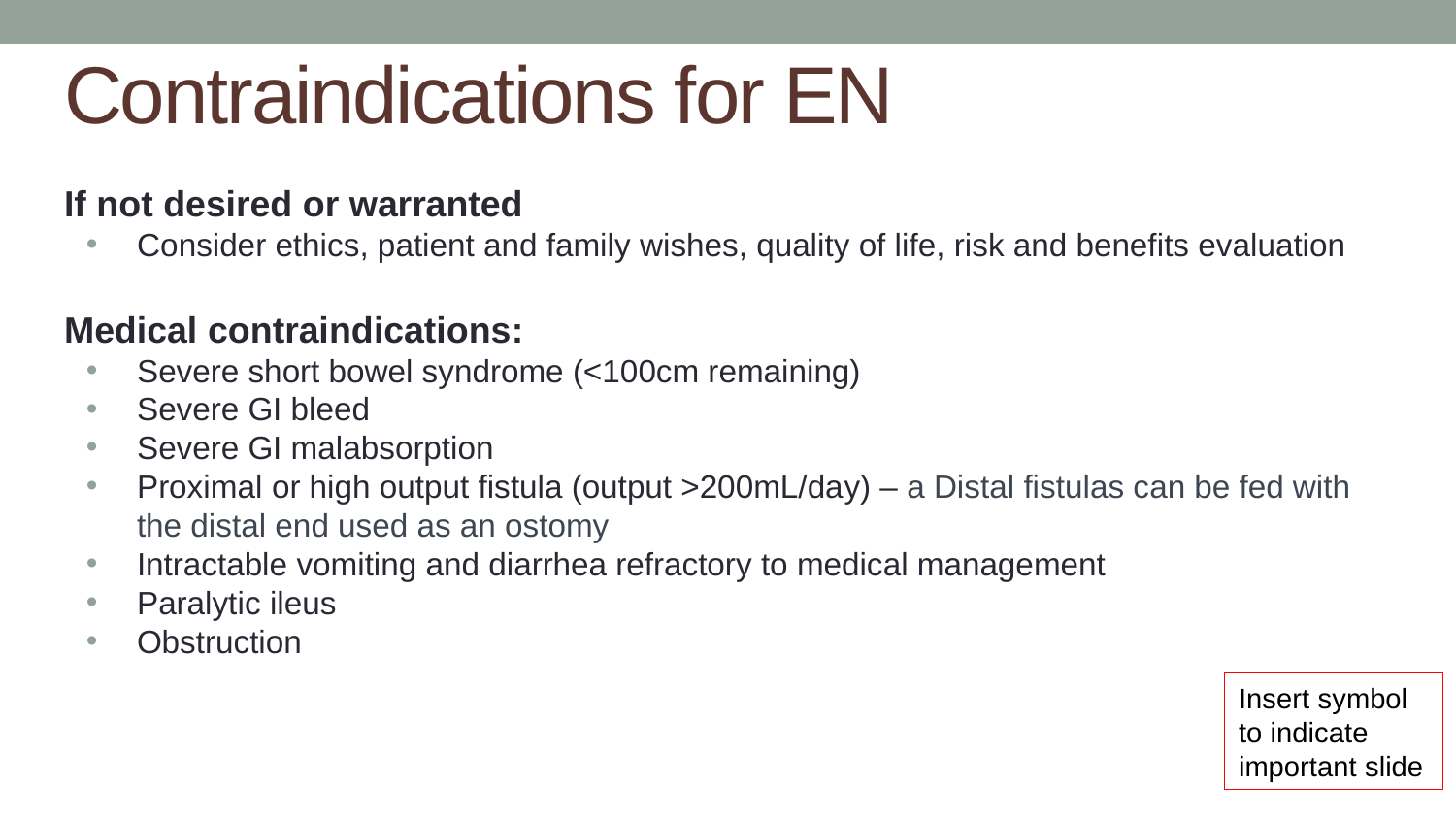

# Contraindications for EN
If not desired or warranted
Consider ethics, patient and family wishes, quality of life, risk and benefits evaluation
Medical contraindications:
Severe short bowel syndrome (<100cm remaining)
Severe GI bleed
Severe GI malabsorption
Proximal or high output fistula (output >200mL/day) – a Distal fistulas can be fed with the distal end used as an ostomy
Intractable vomiting and diarrhea refractory to medical management
Paralytic ileus
Obstruction
Insert symbol to indicate important slide

## Slide 22
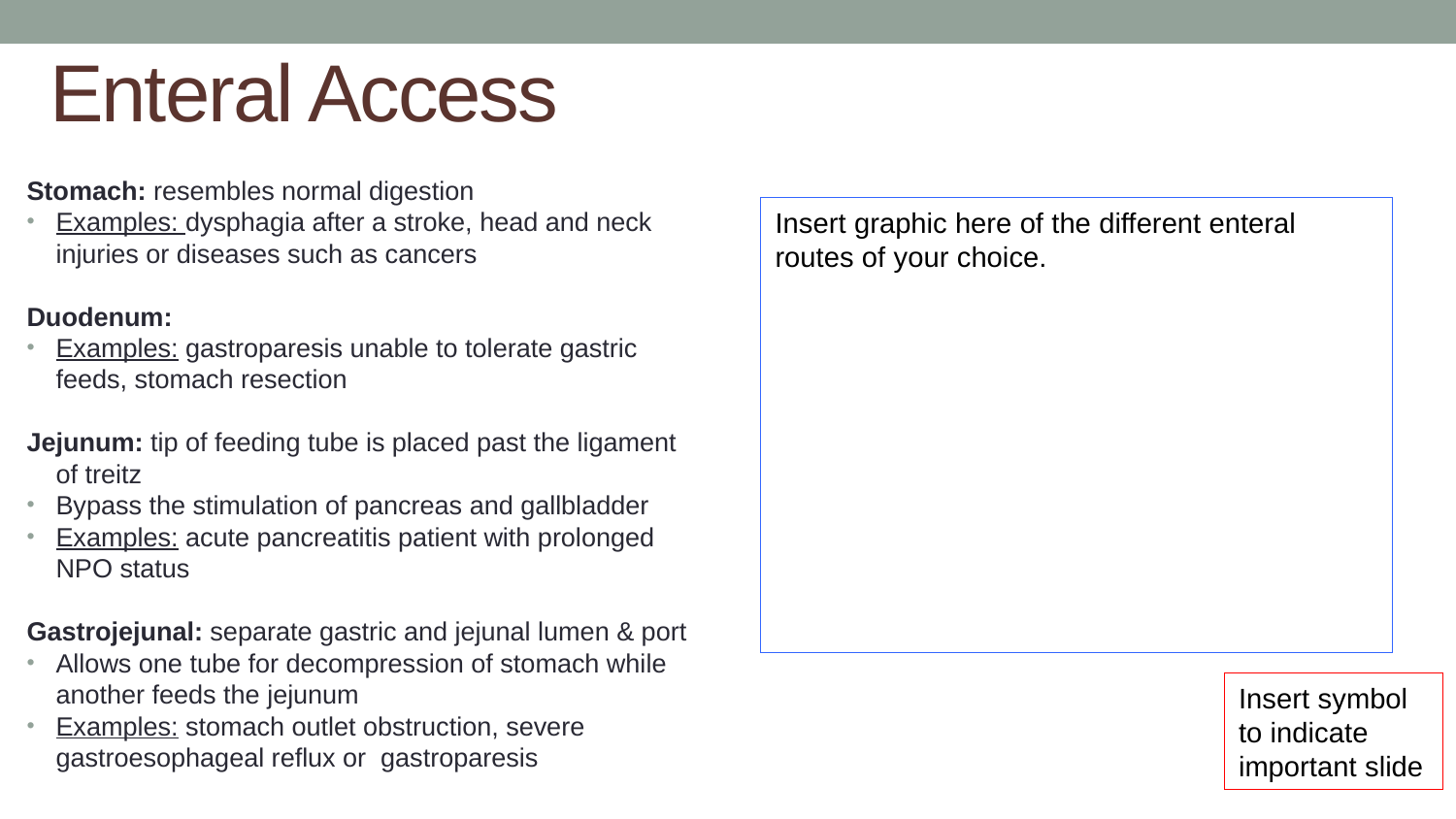

# Enteral Access
Stomach: resembles normal digestion
Examples: dysphagia after a stroke, head and neck injuries or diseases such as cancers
Duodenum:
Examples: gastroparesis unable to tolerate gastric feeds, stomach resection
Jejunum: tip of feeding tube is placed past the ligament of treitz
Bypass the stimulation of pancreas and gallbladder
Examples: acute pancreatitis patient with prolonged NPO status
Gastrojejunal: separate gastric and jejunal lumen & port
Allows one tube for decompression of stomach while another feeds the jejunum
Examples: stomach outlet obstruction, severe gastroesophageal reflux or gastroparesis
Insert graphic here of the different enteral routes of your choice.
Insert symbol to indicate important slide

## Slide 23
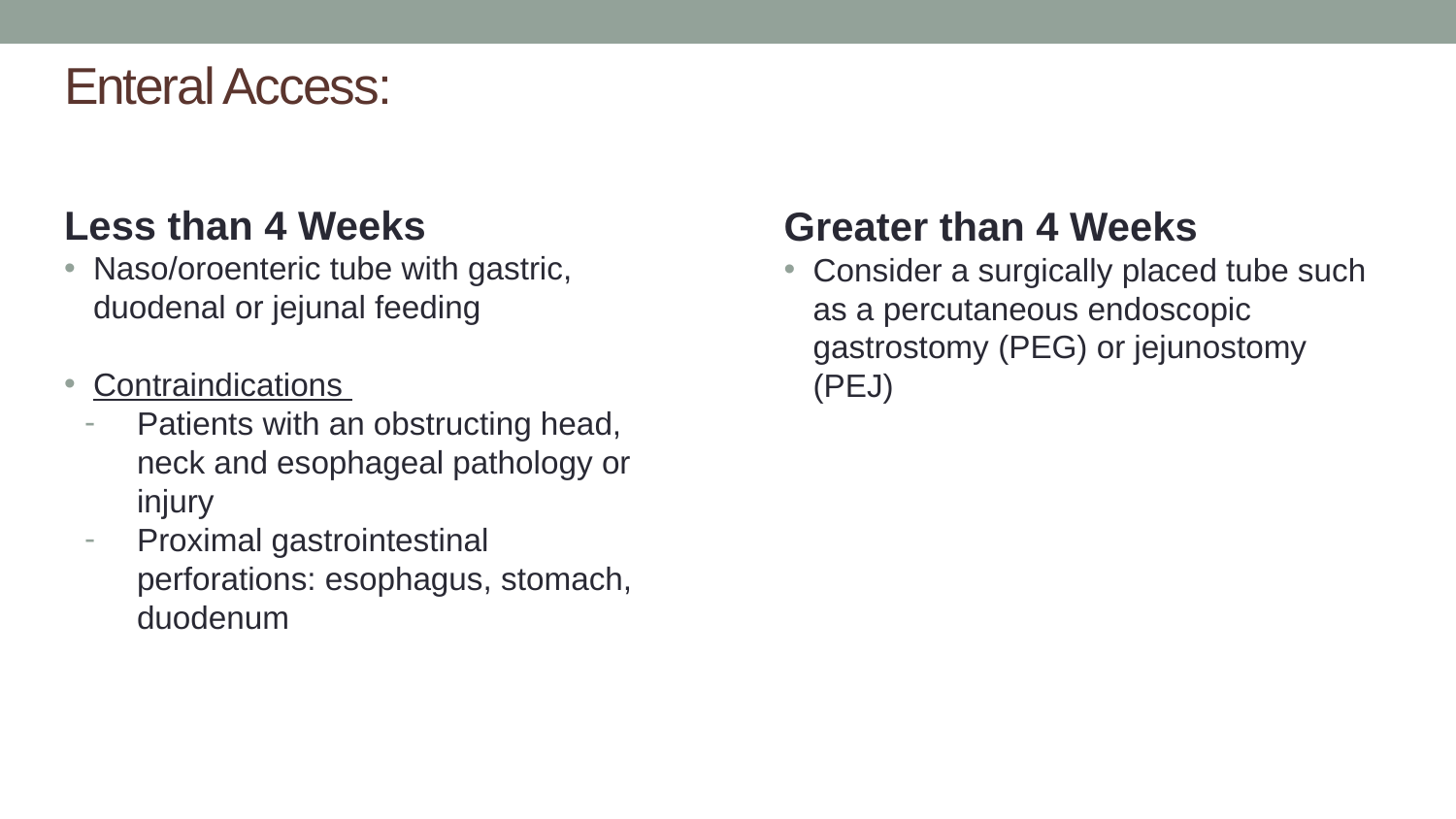

# Enteral Access:
Less than 4 Weeks
Naso/oroenteric tube with gastric, duodenal or jejunal feeding
Contraindications
Patients with an obstructing head, neck and esophageal pathology or injury
Proximal gastrointestinal perforations: esophagus, stomach, duodenum
Greater than 4 Weeks
Consider a surgically placed tube such as a percutaneous endoscopic gastrostomy (PEG) or jejunostomy (PEJ)

## Slide 24
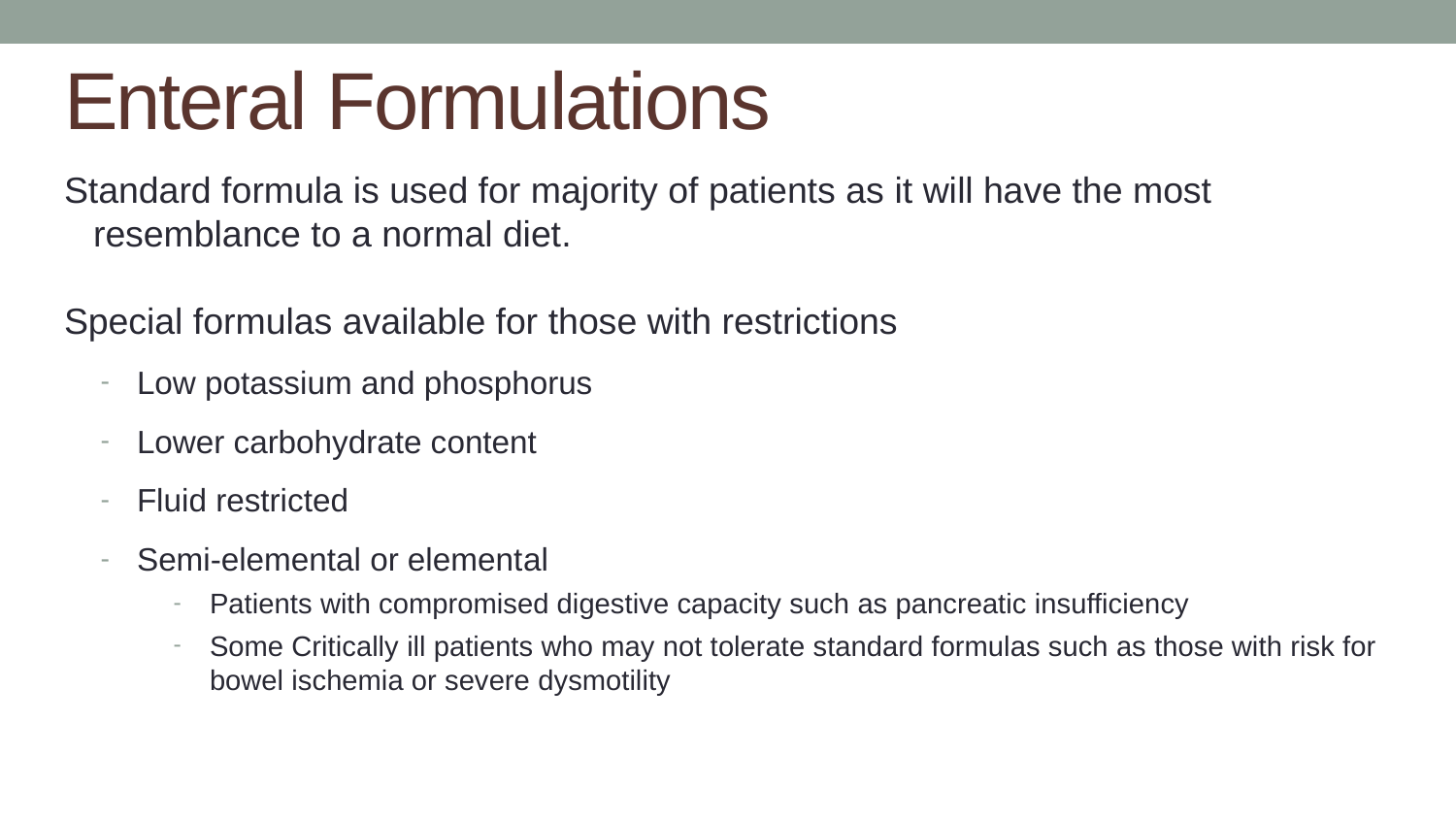

# Enteral Formulations
Standard formula is used for majority of patients as it will have the most resemblance to a normal diet.
Special formulas available for those with restrictions
Low potassium and phosphorus
Lower carbohydrate content
Fluid restricted
Semi-elemental or elemental
Patients with compromised digestive capacity such as pancreatic insufficiency
Some Critically ill patients who may not tolerate standard formulas such as those with risk for bowel ischemia or severe dysmotility

## Slide 25
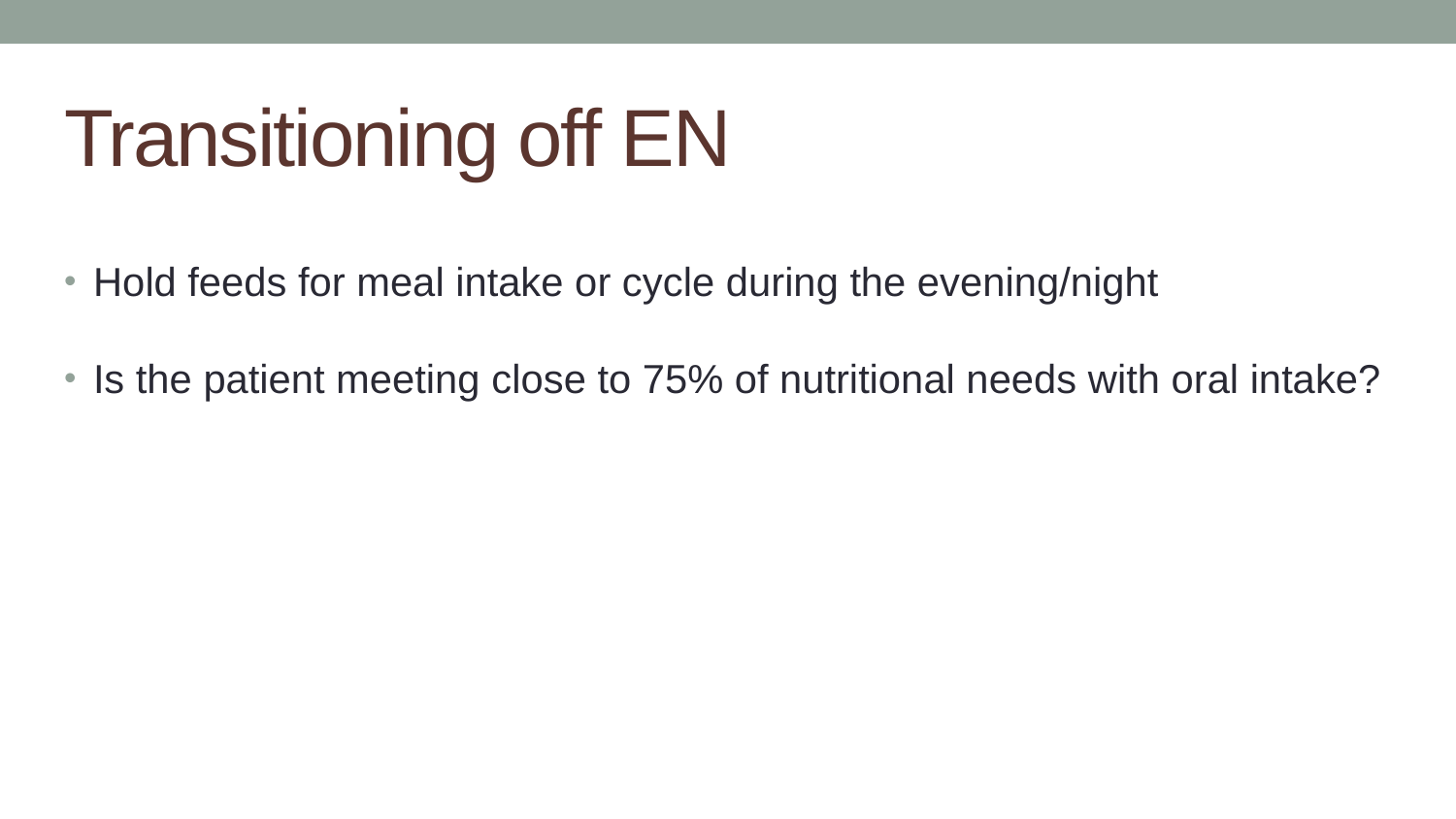

# Transitioning off EN
Hold feeds for meal intake or cycle during the evening/night
Is the patient meeting close to 75% of nutritional needs with oral intake?

## Slide 26
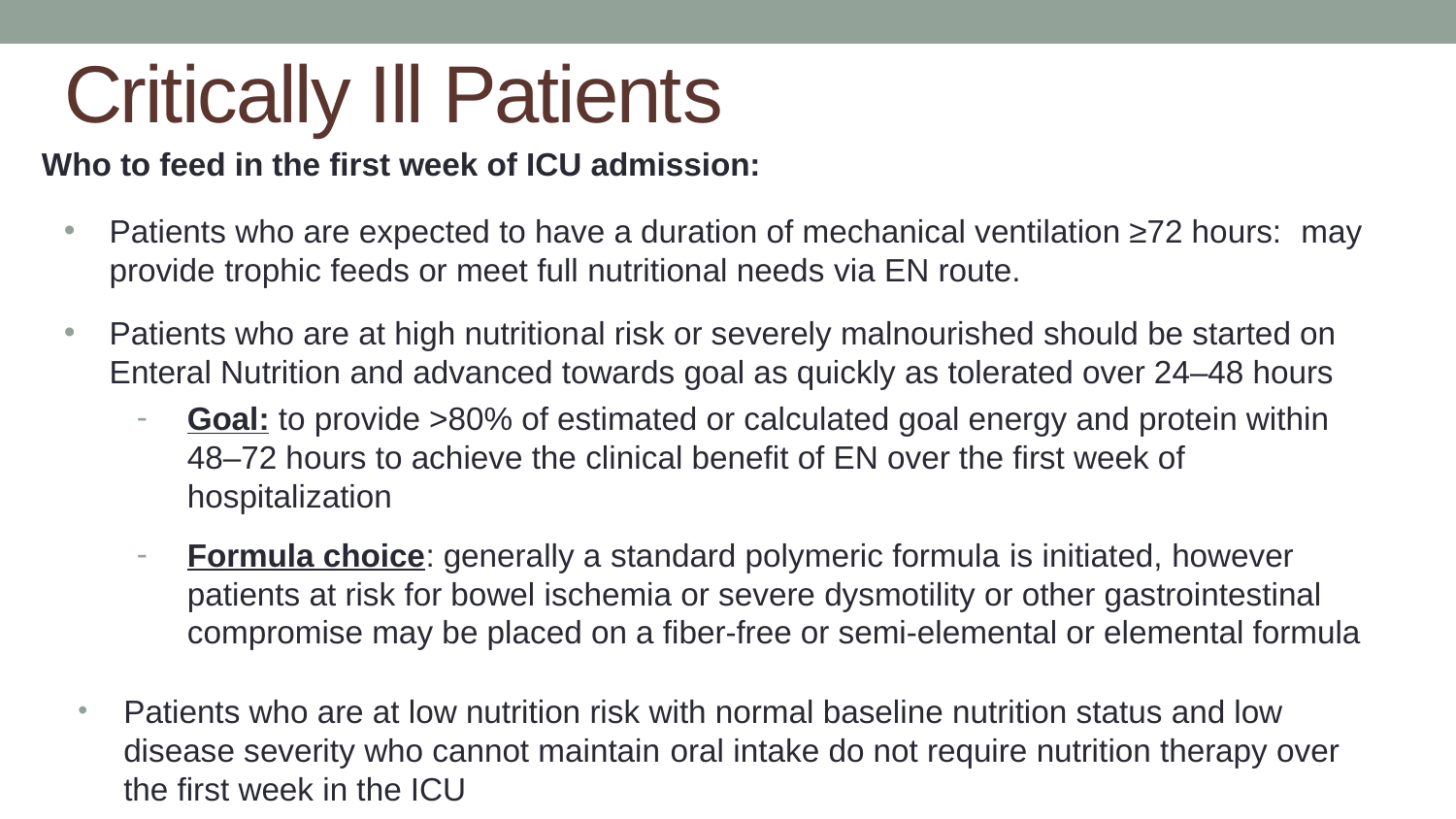

# Critically Ill Patients
Who to feed in the first week of ICU admission:
Patients who are expected to have a duration of mechanical ventilation ≥72 hours: may provide trophic feeds or meet full nutritional needs via EN route.
Patients who are at high nutritional risk or severely malnourished should be started on Enteral Nutrition and advanced towards goal as quickly as tolerated over 24–48 hours
Goal: to provide >80% of estimated or calculated goal energy and protein within 48–72 hours to achieve the clinical benefit of EN over the first week of hospitalization
Formula choice: generally a standard polymeric formula is initiated, however patients at risk for bowel ischemia or severe dysmotility or other gastrointestinal compromise may be placed on a fiber-free or semi-elemental or elemental formula
Patients who are at low nutrition risk with normal baseline nutrition status and low disease severity who cannot maintain oral intake do not require nutrition therapy over the first week in the ICU

## Slide 27
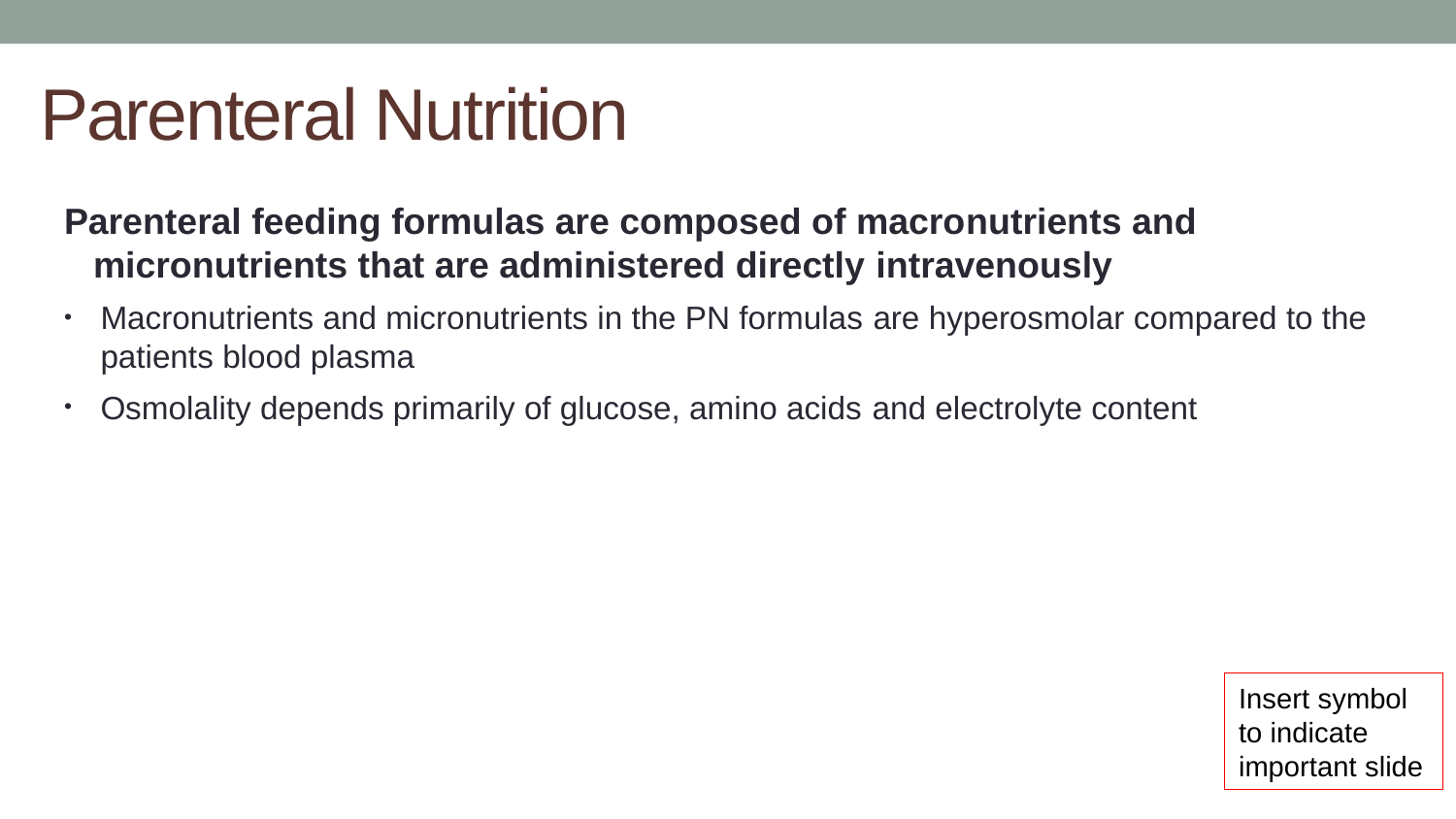

# Parenteral Nutrition
Parenteral feeding formulas are composed of macronutrients and micronutrients that are administered directly intravenously
Macronutrients and micronutrients in the PN formulas are hyperosmolar compared to the patients blood plasma
Osmolality depends primarily of glucose, amino acids and electrolyte content
Insert symbol to indicate important slide

## Slide 28
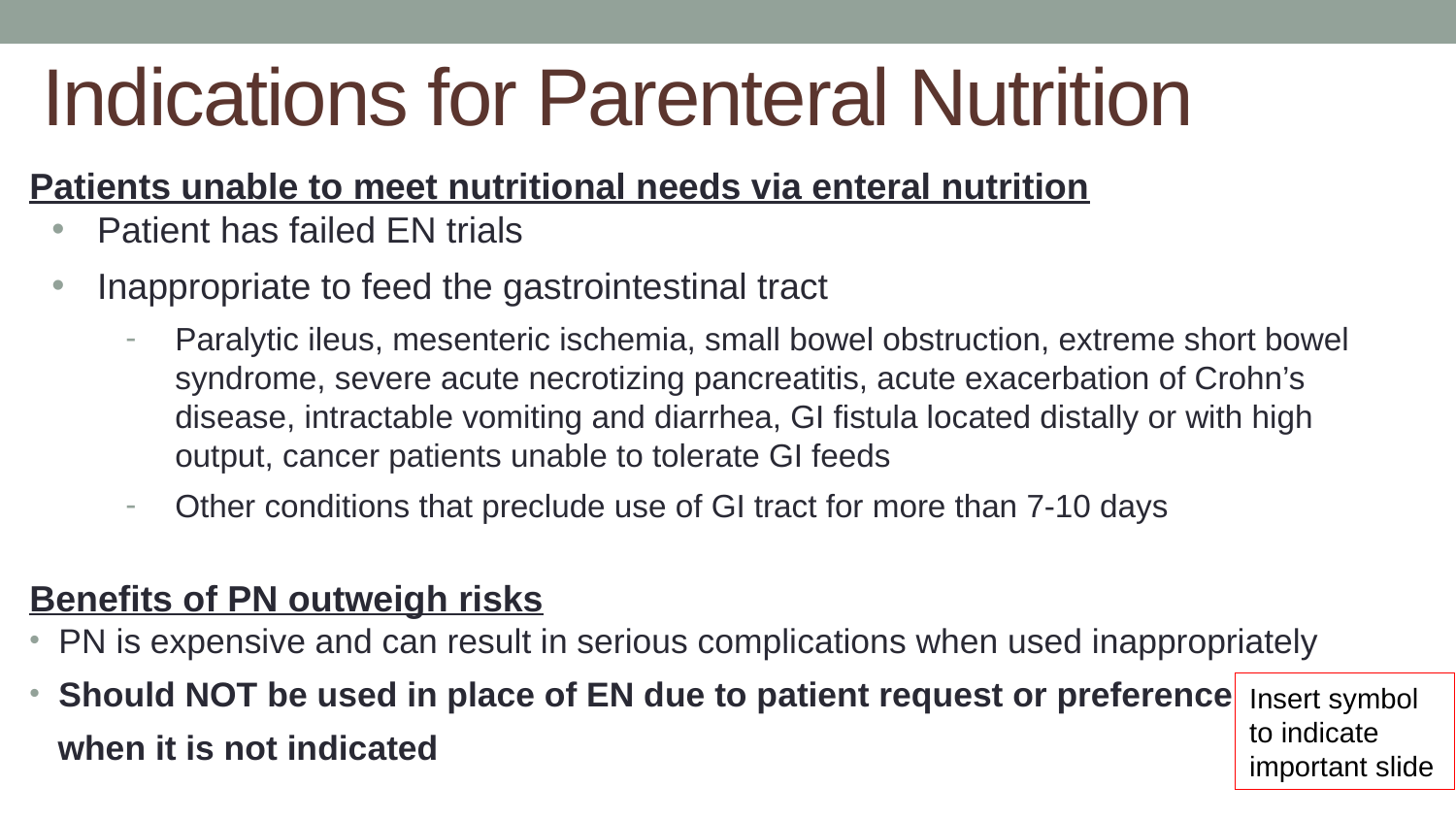

# Indications for Parenteral Nutrition
Patients unable to meet nutritional needs via enteral nutrition
Patient has failed EN trials
Inappropriate to feed the gastrointestinal tract
Paralytic ileus, mesenteric ischemia, small bowel obstruction, extreme short bowel syndrome, severe acute necrotizing pancreatitis, acute exacerbation of Crohn’s disease, intractable vomiting and diarrhea, GI fistula located distally or with high output, cancer patients unable to tolerate GI feeds
Other conditions that preclude use of GI tract for more than 7-10 days
Benefits of PN outweigh risks
PN is expensive and can result in serious complications when used inappropriately
Should NOT be used in place of EN due to patient request or preference
 when it is not indicated
Insert symbol to indicate important slide

## Slide 29
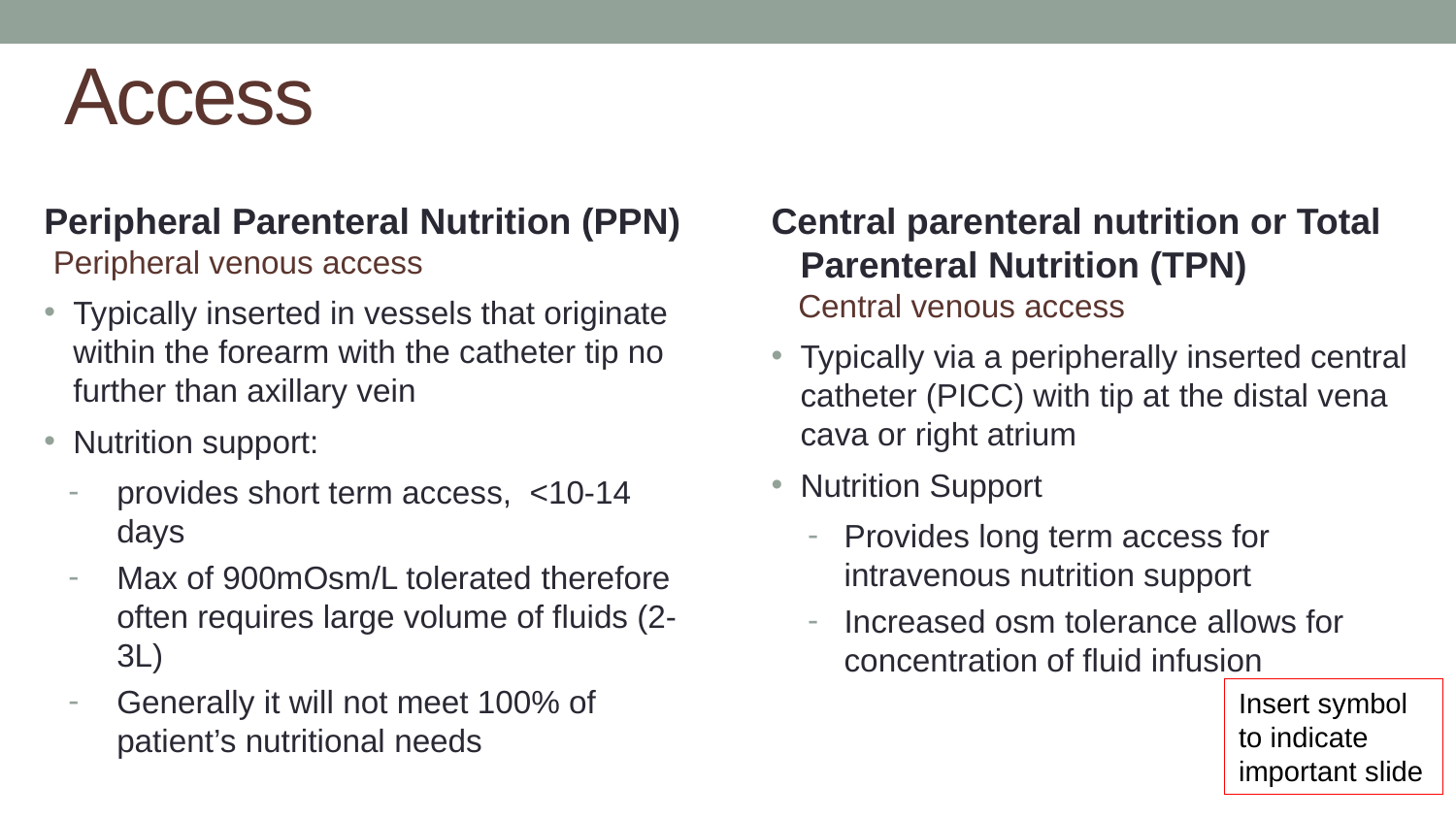

# Access
Peripheral Parenteral Nutrition (PPN)
 Peripheral venous access
Typically inserted in vessels that originate within the forearm with the catheter tip no further than axillary vein
Nutrition support:
provides short term access, <10-14 days
Max of 900mOsm/L tolerated therefore often requires large volume of fluids (2-3L)
Generally it will not meet 100% of patient’s nutritional needs
Central parenteral nutrition or Total Parenteral Nutrition (TPN)
 Central venous access
Typically via a peripherally inserted central catheter (PICC) with tip at the distal vena cava or right atrium
Nutrition Support
Provides long term access for intravenous nutrition support
Increased osm tolerance allows for concentration of fluid infusion
Insert symbol to indicate important slide

## Slide 30
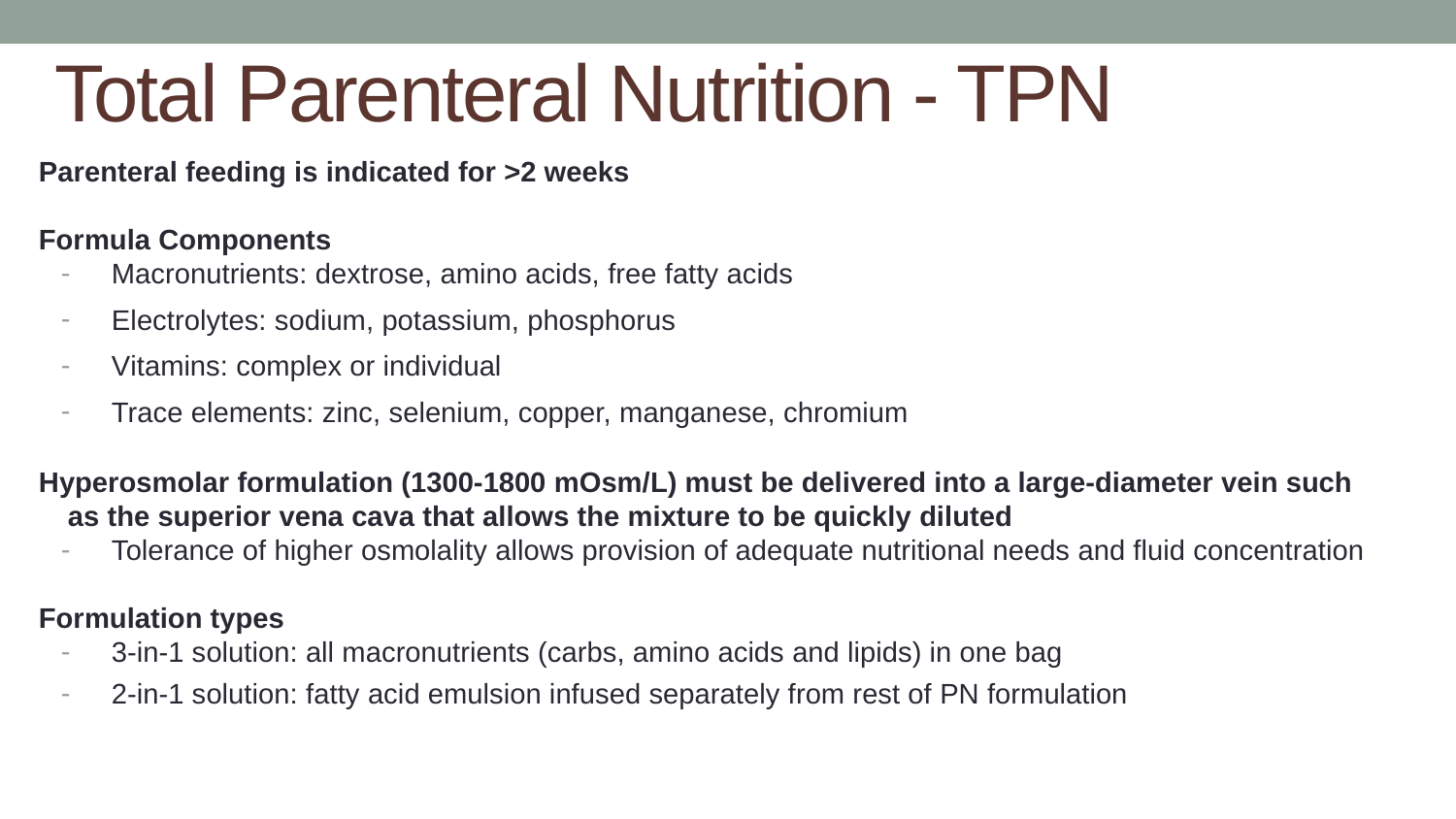

# Total Parenteral Nutrition - TPN
Parenteral feeding is indicated for >2 weeks
Formula Components
Macronutrients: dextrose, amino acids, free fatty acids
Electrolytes: sodium, potassium, phosphorus
Vitamins: complex or individual
Trace elements: zinc, selenium, copper, manganese, chromium
Hyperosmolar formulation (1300-1800 mOsm/L) must be delivered into a large-diameter vein such as the superior vena cava that allows the mixture to be quickly diluted
Tolerance of higher osmolality allows provision of adequate nutritional needs and fluid concentration
Formulation types
3-in-1 solution: all macronutrients (carbs, amino acids and lipids) in one bag
2-in-1 solution: fatty acid emulsion infused separately from rest of PN formulation

## Slide 31
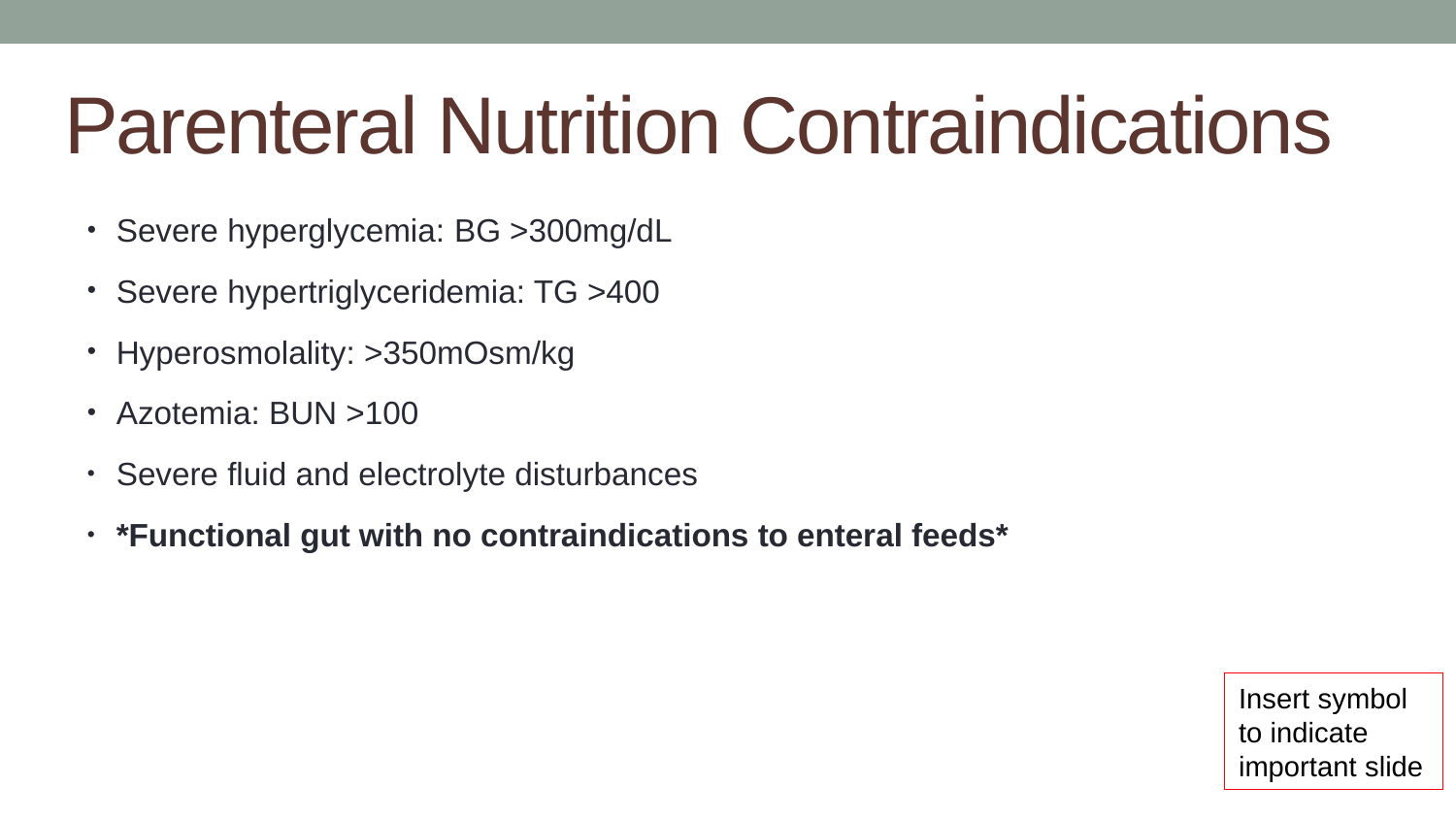

# Parenteral Nutrition Contraindications
Severe hyperglycemia: BG >300mg/dL
Severe hypertriglyceridemia: TG >400
Hyperosmolality: >350mOsm/kg
Azotemia: BUN >100
Severe fluid and electrolyte disturbances
*Functional gut with no contraindications to enteral feeds*
Insert symbol to indicate important slide

## Slide 32
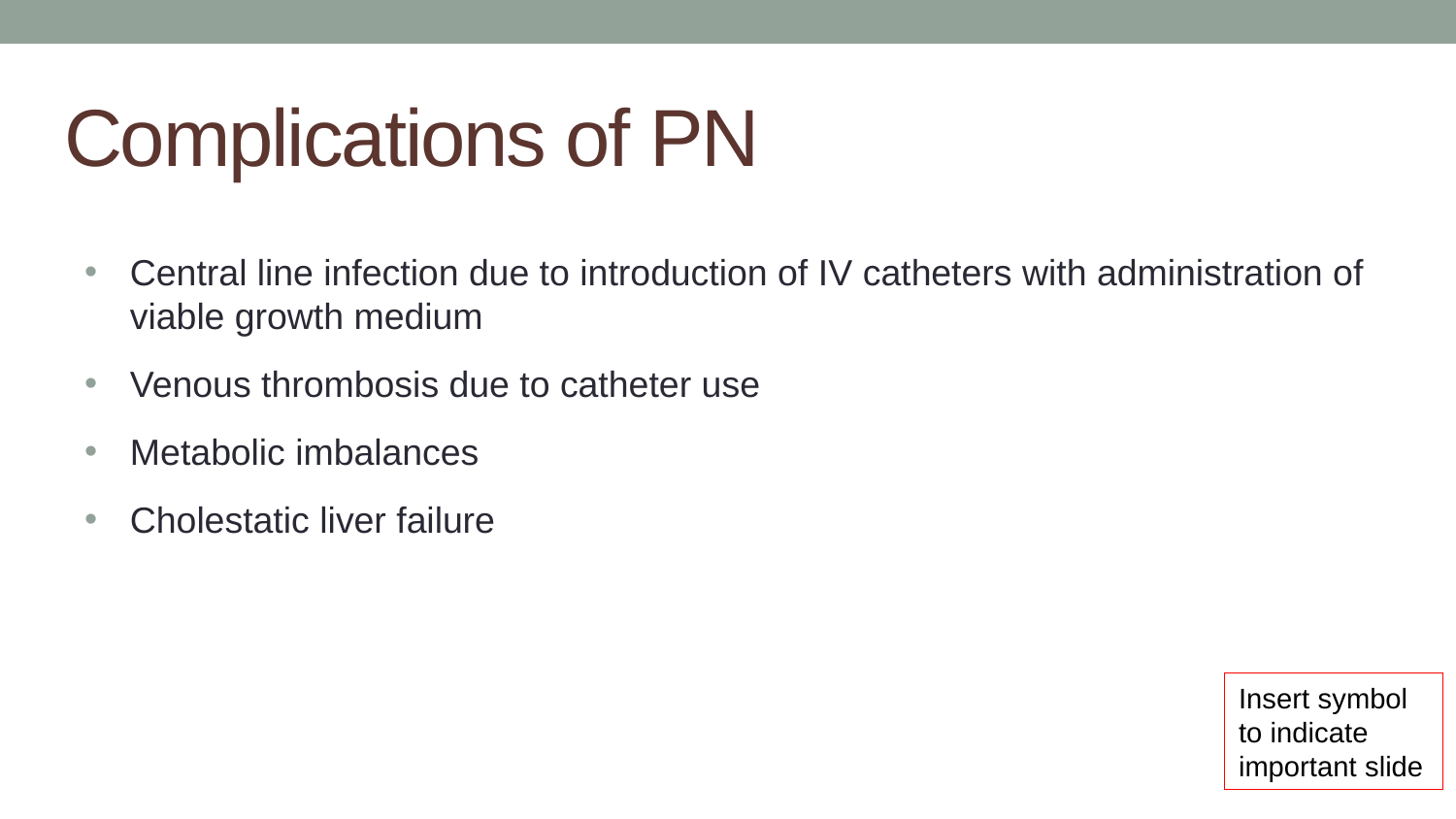

# Complications of PN
Central line infection due to introduction of IV catheters with administration of viable growth medium
Venous thrombosis due to catheter use
Metabolic imbalances
Cholestatic liver failure
Insert symbol to indicate important slide

## Slide 33
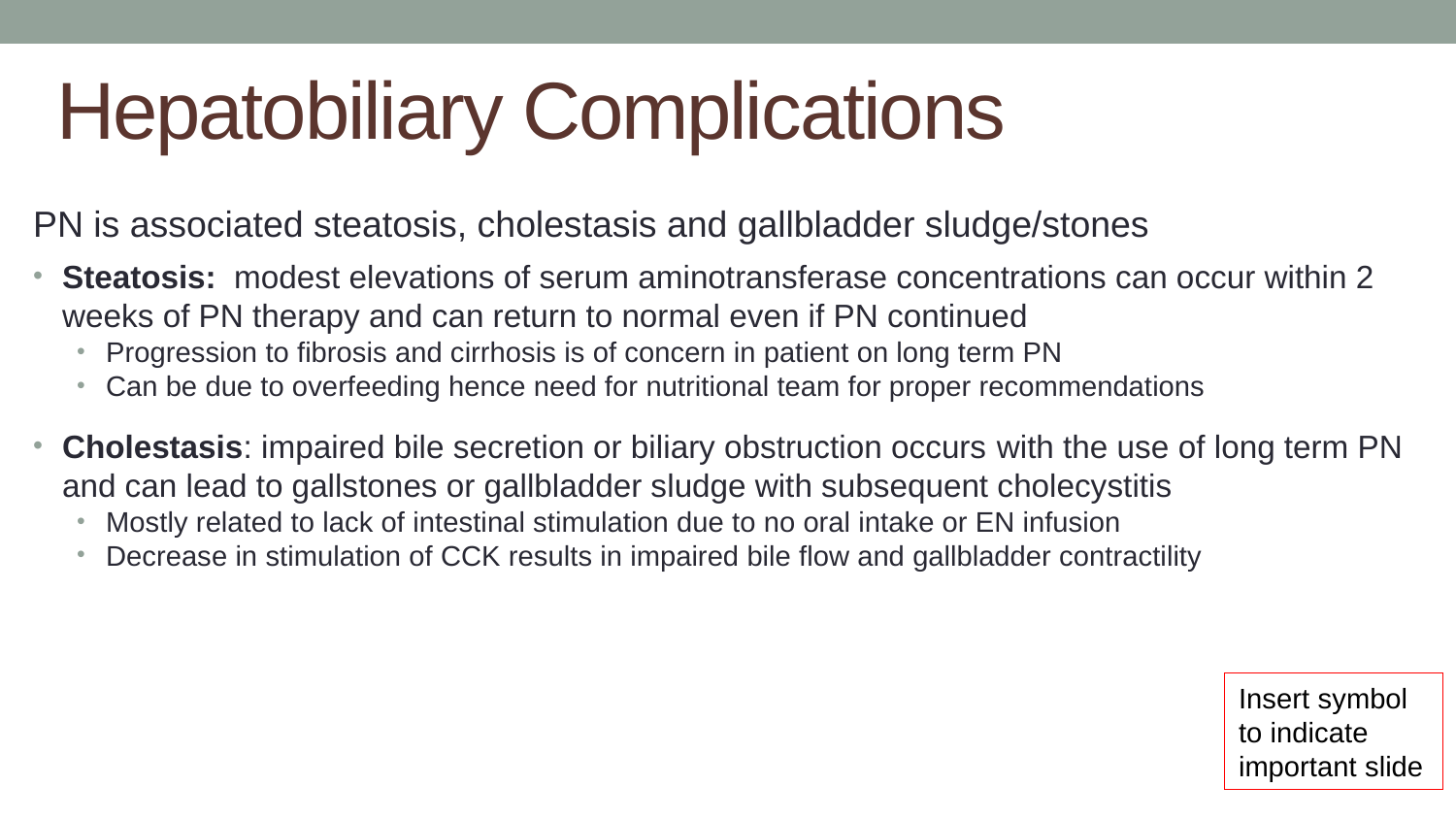

# Hepatobiliary Complications
PN is associated steatosis, cholestasis and gallbladder sludge/stones
Steatosis: modest elevations of serum aminotransferase concentrations can occur within 2 weeks of PN therapy and can return to normal even if PN continued
Progression to fibrosis and cirrhosis is of concern in patient on long term PN
Can be due to overfeeding hence need for nutritional team for proper recommendations
Cholestasis: impaired bile secretion or biliary obstruction occurs with the use of long term PN and can lead to gallstones or gallbladder sludge with subsequent cholecystitis
Mostly related to lack of intestinal stimulation due to no oral intake or EN infusion
Decrease in stimulation of CCK results in impaired bile flow and gallbladder contractility
Insert symbol to indicate important slide

## Slide 34
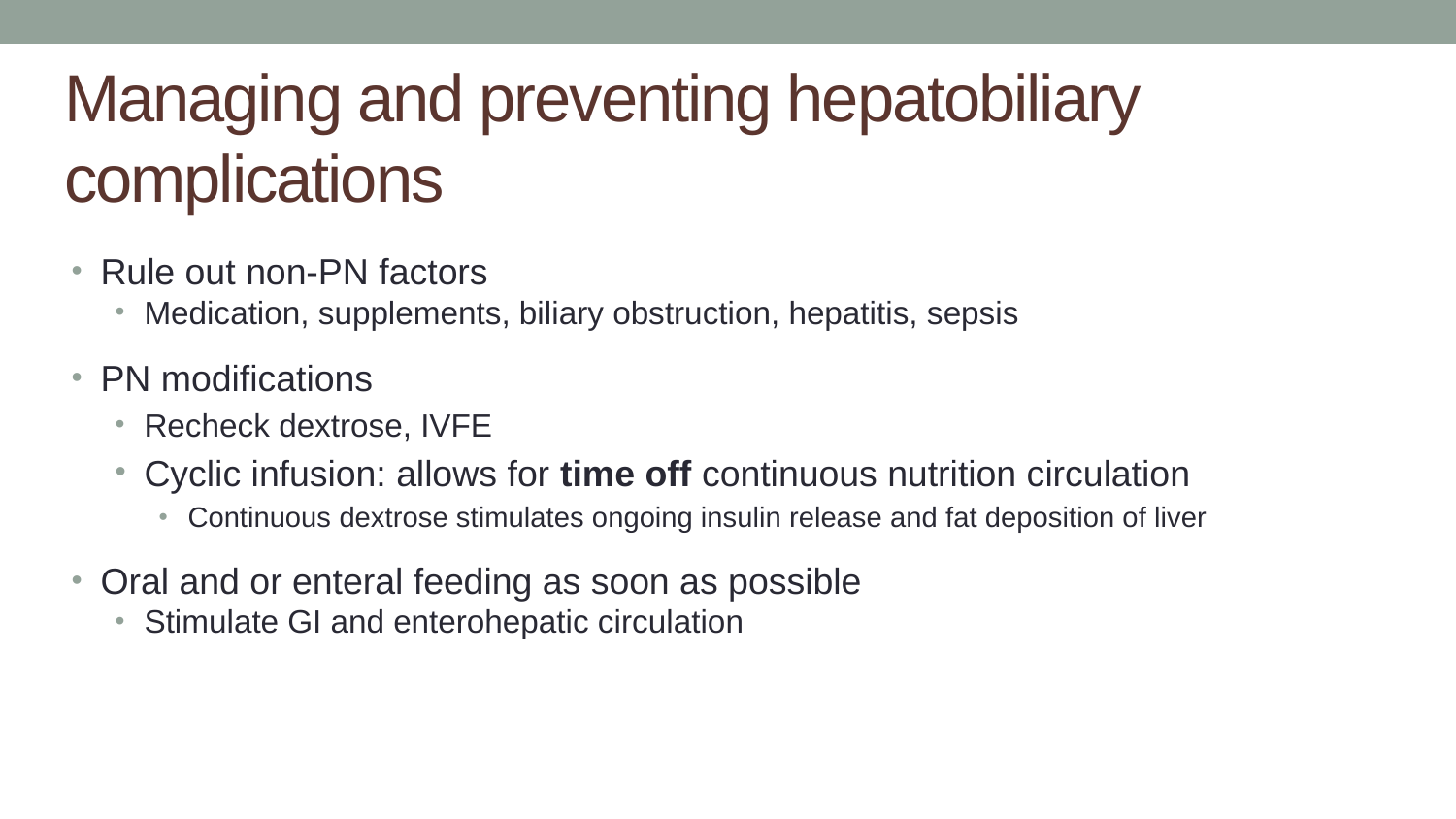

# Managing and preventing hepatobiliary complications
Rule out non-PN factors
Medication, supplements, biliary obstruction, hepatitis, sepsis
PN modifications
Recheck dextrose, IVFE
Cyclic infusion: allows for time off continuous nutrition circulation
Continuous dextrose stimulates ongoing insulin release and fat deposition of liver
Oral and or enteral feeding as soon as possible
Stimulate GI and enterohepatic circulation

## Slide 35
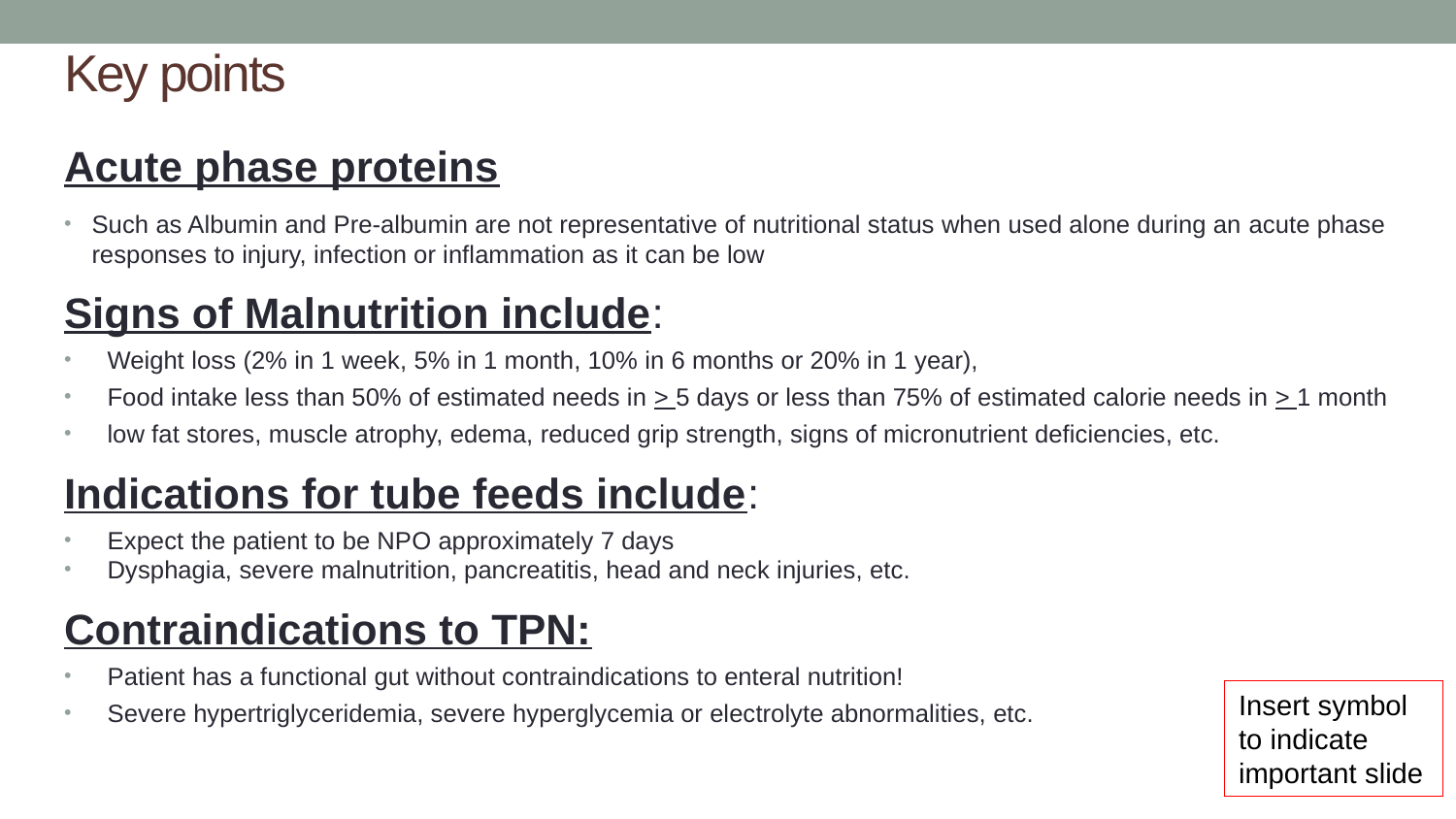

# Key points
Acute phase proteins
Such as Albumin and Pre-albumin are not representative of nutritional status when used alone during an acute phase responses to injury, infection or inflammation as it can be low
Signs of Malnutrition include:
Weight loss (2% in 1 week, 5% in 1 month, 10% in 6 months or 20% in 1 year),
Food intake less than 50% of estimated needs in > 5 days or less than 75% of estimated calorie needs in > 1 month
low fat stores, muscle atrophy, edema, reduced grip strength, signs of micronutrient deficiencies, etc.
Indications for tube feeds include:
Expect the patient to be NPO approximately 7 days
Dysphagia, severe malnutrition, pancreatitis, head and neck injuries, etc.
Contraindications to TPN:
Patient has a functional gut without contraindications to enteral nutrition!
Severe hypertriglyceridemia, severe hyperglycemia or electrolyte abnormalities, etc.
Insert symbol to indicate important slide

## Slide 36
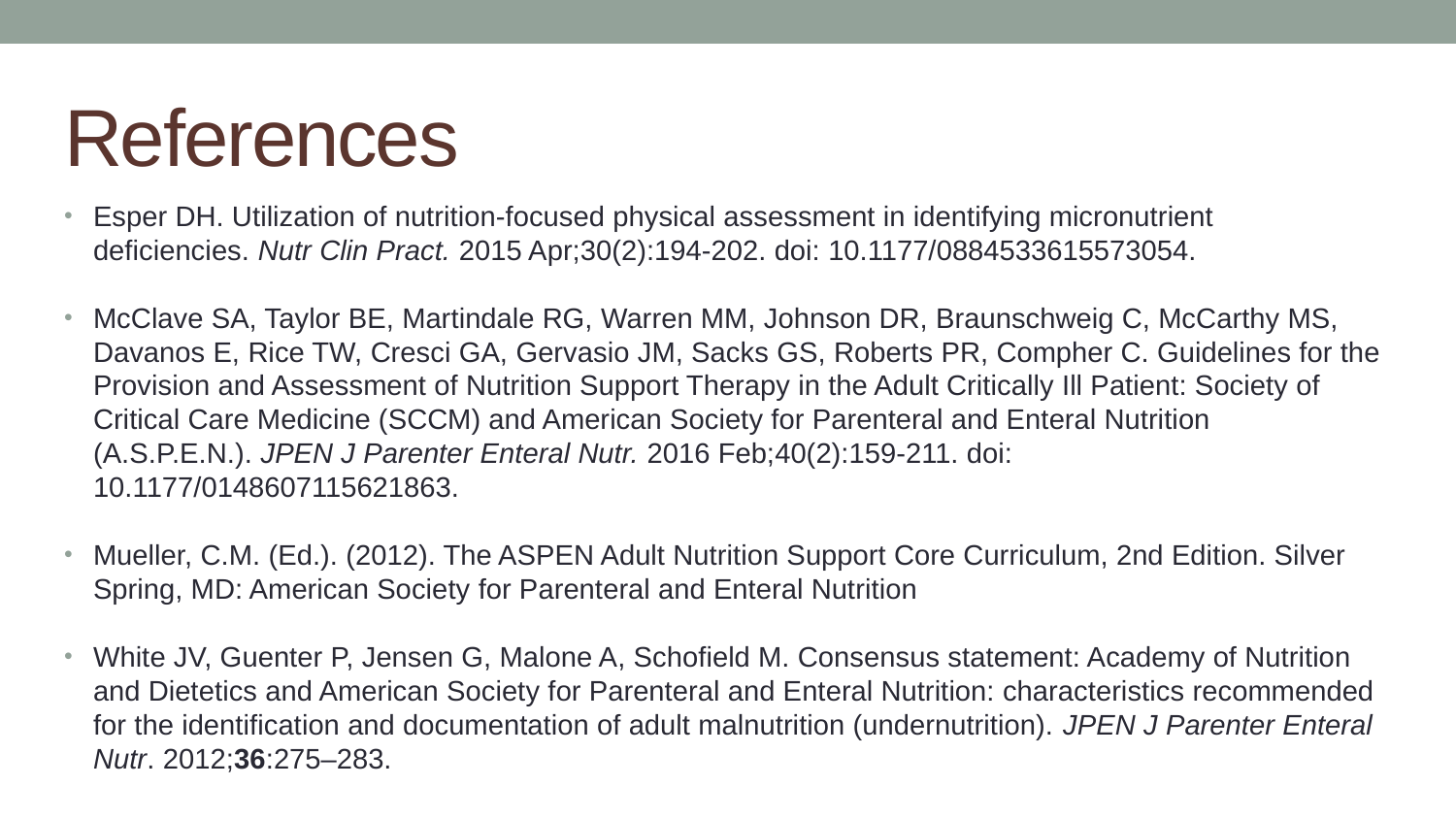

# References
Esper DH. Utilization of nutrition-focused physical assessment in identifying micronutrient deficiencies. Nutr Clin Pract. 2015 Apr;30(2):194-202. doi: 10.1177/0884533615573054.
McClave SA, Taylor BE, Martindale RG, Warren MM, Johnson DR, Braunschweig C, McCarthy MS, Davanos E, Rice TW, Cresci GA, Gervasio JM, Sacks GS, Roberts PR, Compher C. Guidelines for the Provision and Assessment of Nutrition Support Therapy in the Adult Critically Ill Patient: Society of Critical Care Medicine (SCCM) and American Society for Parenteral and Enteral Nutrition (A.S.P.E.N.). JPEN J Parenter Enteral Nutr. 2016 Feb;40(2):159-211. doi: 10.1177/0148607115621863.
Mueller, C.M. (Ed.). (2012). The ASPEN Adult Nutrition Support Core Curriculum, 2nd Edition. Silver Spring, MD: American Society for Parenteral and Enteral Nutrition
White JV, Guenter P, Jensen G, Malone A, Schofield M. Consensus statement: Academy of Nutrition and Dietetics and American Society for Parenteral and Enteral Nutrition: characteristics recommended for the identification and documentation of adult malnutrition (undernutrition). JPEN J Parenter Enteral Nutr. 2012;36:275–283.
