# Supplementary material for: Nutrition Education on the Wards: A Self-Study Module for Improving Medical Student Knowledge of Nutrition Assessment and Interventions
Source: MedEdPORTAL. 2020 Oct 16;16:10968. doi: 10.15766/mep_2374-8265.10968 (PMC7566223; doi:10.15766/mep_2374-8265.10968)
Supplement: Supplementary file 1 — Instructions.docxPremodule Nutrition Evaluation Survey.docxNutrition Education Module.pptxPostmodule Nutrition Evaluation Survey.docxAnswer Key.docx [file mep_2374-8265.10968-s001.zip › D. Postmodule Nutrition Evaluation Survey.docx]

**Appendix D**

**Nutrition Education on the Wards: Post-Module Survey**

1. Which clerkship have you just completed?

- Surgery
- Medicine
- Subinternship in Internal Medicine
- Critical Care

1. Have you had any formal nutrition education during this rotation (medicine or surgery at RWJUH) aside from the Nutrition Support Introduction Module?

- Yes
- No

1. Have you come across any patient case that required nutrition intervention with a nutrition consult?

- Yes
- No

1. Did the module help you feel more comfortable with cases that require nutrition intervention, such as understanding the need for a nutrition intervention or knowing when to consult the nutrition team?

- Extremely comfortable
- Moderately comfortable
- Neither comfortable nor uncomfortable
- Moderately uncomfortable
- Extremely uncomfortable

1. I have an understanding of basic nutritional requirements such as how to determine general calories needs and macronutrients.

- I do not
- I can state the 3 main macronutrients

1. I understand the role of acute phase serum protein such as albumin in interpreting a patient’s nutritional status

- No
- I know what an acute phase protein is but not how to use it in nutrition assessment.
- Yes

1. I can state at least 2 factors that qualifies a patients to be malnourished

- No
- I can name 1
- Yes

1. I understand the difference between enteral and parenteral nutrition.

- No
- Yes
- I can only describe enteral nutrition
- I can only describe parenteral nutrition

1. I can state 3 reasons why a patient may need enteral feeds

- No
- I can name 1 or 2 reasons
- Yes

1. I can state 3 contraindications for TPN

- No
- I can name 1 or 2 contraindication
- Yes

1. How do you rate the overall value of this module?

- I learned new information that was helpful in completing patient assessment during my rotation.
- I learned new information, however a nutrition evaluation was not completed or necessary for the patient assessments I participated in during my rotation.
- The content of this module was not relevant to my rotation.

1. Please provide any additional comments you may have regarding this module for future improvement:

**Page Break (student must flip page or click arrow to get to the next set of questions, if this is done on qualtrics)**

1. Please state an acute phase protein and its use in nutrition assessment:
2. State at least 2 factors that qualify a patients to be malnourished:
3. State the difference between enteral and parenteral nutrition:
4. State 3 reasons why a patient may need enteral feeds:
5. State 3 contraindications for TPN:
