# Supplementary material for: Nutrition Education on the Wards: A Self-Study Module for Improving Medical Student Knowledge of Nutrition Assessment and Interventions
Source: MedEdPORTAL. 2020 Oct 16;16:10968. doi: 10.15766/mep_2374-8265.10968 (PMC7566223; doi:10.15766/mep_2374-8265.10968)
Supplement: Supplementary file 1 — Instructions.docxPremodule Nutrition Evaluation Survey.docxNutrition Education Module.pptxPostmodule Nutrition Evaluation Survey.docxAnswer Key.docx [file mep_2374-8265.10968-s001.zip › E. Answer Key.docx]

**Appendix E**

**Nutrition Education on the Wards:**

**Answer Key for the Open-ended Questions**

1. Please state an acute phase protein and its use in nutrition assessment:
   1. Albumin and pre-albumin are acute phase proteins that may be low during an acute phase response such as injury, infection or inflammation.
   2. Acute phase proteins such as albumin and pre-albumin are not representative of nutritional status when used alone
2. State at least 2 factors that qualify a patients to be malnourished:
   1. Intake < 75% of estimated energy requirement for > 1 month
   2. Intake < 50% of estimated energy requirement for > 5 days
   3. Weight loss > 2 % in 1 week
   4. Weight loss > 5% in 1 month
   5. Weight loss > 7.5% in 3 months
   6. Weight loss > 10% in 6 months
   7. Weight loss > 20 % in 1 year
   8. Muscle mass loss
   9. Loss of subcutaneous fat
   10. Fluid accumulation
   11. Reduced grip strength
3. State the difference between enteral and parenteral nutrition:
   1. Enteral Nutrition is nutrition formulas that are provided through the gastrointestinal tract via a tube, catheter or stoma that delivers nutrients distal to the mouth
   2. Parenteral nutrition is nutrition formulas that are provided directly intravenously
4. State 3 reasons why a patient may need enteral feeds:
   1. Expect the patient to be NPO approximately 7 days
   2. Disease examples: Dysphagia, gastroparesis, severe malnutrition, pancreatitis, head and neck injuries or diseases
5. State 3 contraindications for TPN:
   1. Patient has a functional gut without contraindications to enteral nutrition
   2. Hyperosmolality
   3. Severe hypertriglyceridemia
   4. Severe hyperglycemia
   5. Electrolyte abnormalities
   6. Volume overload
