# Supplementary material for: Nutrition Education on the Wards: A Self-Study Module for Improving Medical Student Knowledge of Nutrition Assessment and Interventions
Source: MedEdPORTAL. 2020 Oct 16;16:10968. doi: 10.15766/mep_2374-8265.10968 (PMC7566223; doi:10.15766/mep_2374-8265.10968)
Supplement: Supplementary file 1 — Instructions.docxPremodule Nutrition Evaluation Survey.docxNutrition Education Module.pptxPostmodule Nutrition Evaluation Survey.docxAnswer Key.docx [file mep_2374-8265.10968-s001.zip › _Educational_Summary_Report_10968.pdf]

MedEdPORTAL® The AAMC Journal of Teaching and Learning Resources

Original Publication Open Access

Nutrition Education on the Wards: A Self-Study Module for Improving Medical Student Knowledge of Nutrition Assessment and Interventions

Barbara Dutra, MD, RD*, Matthew Lissauer, MD, Hanin Rashid, PhD, EdM

*Corresponding Author: bdutra07@gmail.com

Abstract

Introduction: Nutrition plays a key role in the prevention and treatment of disease. Hospitalized patients are often malnourished, which is a major contributor to medical complications, decreased quality of life, lengthened medical stay, increased health care costs, and mortality. However, medical students continue to have inadequate education in nutrition and report feeling poorly trained in nutrition. We proposed an online module that could be used by medical students as a self-study activity to learn about key signs for the diagnosis of malnutrition and the nutrition interventions available in the hospital setting. **Methods:** Third- and fourth-year medical students at Rutgers Robert Wood Johnson Medical School in medicine, surgery, and critical care clerkships were given access to an online nutrition education module discussing the signs of malnutrition in hospitalized patients and the interventions available in the inpatient setting. A premodule and postmodule survey was given via email at the beginning and at the end of the clerkship. A one-sample *t* test was used to assess the relationship between the mean scores of the pre- and postmodule surveys. **Results:** One hundred nine out of 255 students responded to the premodule survey. Thirty-two students completed the module and postmodule survey. There was a significant difference in mean scores between students who completed the module and postmodule survey compared to the overall student population prior to having access to the module. **Discussion:** Medical students have limited training in nutrition education, and our findings show that a self-study online module can improve students' knowledge.

Keywords
Nutrition, Inpatient, Nutrition Support, Enteral, Parenteral, Malnutrition, Self-Study, Online/Distance Learning, Self-Regulated Learning, Virtual Learning

Educational Objectives

By the end of this module, learners will be able to:

1. State factors related to malnutrition.
2. Recognize the role of a registered dietitian in a multidisciplinary team.
3. Describe the different types of nutrition support available to patients.
4. Identify indications and contraindications for the different nutrition support modalities.

Introduction

The actual prevalence of malnutrition in the hospital setting is unknown due to the use of different criteria to diagnose malnutrition. However, since the 1970s, studies have reported that the prevalence of malnutrition in the hospital setting ranged from 20% to 62%.[1-4] While dietary intake has been shown to be amongst the leading risk factors of disease burden,[5] there continues to be a lack of training in nutrition amongst medical students and physicians. We present here an overview of the impact of malnutrition in the hospital setting, as well as the need for training in nutrition amongst medical students, and propose a module that is simple to implement and informative for medical students.

Citation:
Dutra B, Lissauer M, Rashid H. Nutrition education on the wards: a self-study module for improving medical student knowledge of nutrition assessment and interventions. *MedEdPORTAL.* 2020;16:10968. https://doi.org/10.15766/mep_2374-8265.10968

The Impact of Malnutrition

Malnutrition syndromes in the clinical setting have been defined by experts on an international guideline committee as starvation-related malnutrition, chronic disease–related malnutrition, acute disease, or injury-related malnutrition.[6] Experts have also developed a set of characteristics to assist in the detection and diagnosis of malnutrition, including insufficient energy intake, weight loss, and loss of muscle mass.[7] Poor nutritional status has been associated with worse outcomes, from impaired immunity to

Copyright © 2020 Dutra et al. This is an open-access publication distributed under the terms of the Creative Commons Attribution license.

increased risk of infections, cardiac and pulmonary complications, decreased functional status and wound healing, and pressure ulcers.[1,4,7-13] Ultimately, malnutrition has been shown to be an independent risk factor not only for poor outcomes but also for increased length of hospital stay, costs, and mortality.[4,14,15] Since the incorporation of malnutrition screening, the provision of nutrition support to the malnourished hospitalized patient has been shown to decrease length of hospital stay, decrease readmission rates, and decrease costs.[16-21]

The Need for Medical Nutrition Education

Despite the extensive data reporting on the importance of nutrition in disease prevention and management, there has been an ongoing deficit in nutrition education for physicians, including in medical school training.[22-26] A review of the literature by Frantz, Munroe, McClave, and Martindale from the 1950s to 2011 found that medical students and physicians in the United States reported poor training in nutrition.[24] Given the lack of nutrition knowledge amongst physicians in training and practice, the National Academy of Science recommended at least 25 hours of nutrition education in graduate medical school.[27] To meet these requirements, multiple efforts have been made to improve nutrition education in medical training. The National Heart, Lung, and Blood Institute and the National Institute of Diabetes and Digestive and Kidney Diseases established the Nutrition Academic Award (NAA) Program that provided funding from 1998 to 2005 to 21 US medical schools to strictly support nutrition education in medical curricula.[28,29] Other institutions, such as the University of North Carolina, developed a standardized curriculum in the 1990s that remains available to all medical schools as a computerized teaching tool.[30,31]

By 2010, Adams, Kohlmeier, and Zeisel found that out of 83% (105) of accredited US medical schools between 2008 and 2009, medical students received on average 19.6 hours of nutrition education; only 27% of the schools provided the minimum of 25 hours recommended by the National Academy of Science, and only 25% required a dedicated nutrition course.[25] Due to the ongoing deficit in nutrition training, the NAA developed the *Nutrition Curriculum Guide for Training Physicians* with over 200 educational learning objectives for medical students, residents, and physicians.[32] Yet a recent survey in 2018 again reported that while medical students believe physicians have an important role in the provision of nutrition care to patients, the students felt they have inadequate training in nutrition.[26] The ongoing lack of adequate training in nutrition is likely multifactorial in the setting of already extensive curriculum requirements, lower than recommended hours of nutrition education, lack of or limited effectiveness of established curricula, lack of or limited availability of educators with expertise in nutrition, and lack of priority to nutrition education.[23,26,33]

A Proposal for Nutrition Education for the Wards

The Liaison Committee on Medical Education (LCME) recommends that medical schools incorporate self-directed learning activities into their curricula to promote lifelong learning skills.[34] Given the aforementioned barriers to nutrition training, this offers an opportunity to implement a self-study learning activity with medical students during their clinical years.[35,36] Nutrition education ranges from the biochemical function of nutrients to clinical interventions. We created and implemented a self-study nutrition education tool that can be used by medical students during their clinical years. The goal for this tool was to provide clinically relevant nutrition education on the signs for the diagnosis of malnutrition, as well as on the interventions available in the inpatient setting, and to assess the effectiveness of independent study of nutrition by medical students. The module was not meant to provide medical students with extensive training by registered dietitians but instead aimed to help students recognize the role of nutrition and the available modalities in a short and succinct format keeping in mind the extensive medical training received during medical school. Our module complemented other tools on nutrition education for hospitalized patients. Chandra and colleagues provided a set of tools to be used by instructors to educate medical students in a class format[37]; Elder, Perone, Branski, and Brown developed an in-class education module to teach medical students the role of nutrition in critically ill patients[38]; and Ray and colleagues demonstrated that a 1-day workshop was effective at educating medical students in nutrition topics, including malnutrition.[39] Our module was created with the intention to provide the most essential information that medical students ought to understand and could study during their own independent study time to overcome barriers of limited faculty availability.

Methods

The module and survey were developed by one of the authors, who, at the time of the study design, was a medical student as well as a registered dietitian and certified nutrition support clinician. The content of the module was developed to address the data supporting the complications of malnutrition by teaching medical students during their clinical years the role of nutrition support in the hospital setting. The module reviewed aspects of a nutrition assessment, signs qualifying a patient for the diagnosis of malnutrition, and the interventions available to manage and prevent malnutrition in the hospital, including a review of enteral

Copyright © 2020 Dutra et al. This is an open-access publication distributed under the terms of the Creative Commons Attribution license.

and parenteral nutrition support. A 1-month pilot was done with a more extensive PowerPoint; however, from verbal feedback that the module was too long and detailed, the module was modified to contain key learning points for medical students that could be reviewed in approximately 30 minutes. Facilitators were provided with an instructor's guide (Appendix A) to aid in the module's implementation.

Third- and fourth-year medical students in internal medicine, surgery, and critical care clerkships at Rutgers Robert Wood Johnson Medical School (RRWJMS) were invited between August 2017 and July 2018 to review the online module and complete a premodule and postmodule survey. An email sent through Qualtrics invited medical students to participate in the premodule survey (Appendix B) at the start of their clerkship. The first page of the premodule survey was a consent form. If students completed the survey, the nutrition education module (Appendix C) was accessible via a link at the end of the initial survey at the start of the clerkships. Students could then use the module as a reference tool throughout their clerkship and beyond. Finally, a postmodule survey (Appendix D) was sent via email through Qualtrics at the end of the clerkship. An answer key (Appendix E) to the open-ended questions in the survey was provided for facilitators. Students were allowed to withdraw from the study at any point in time. On the last page of the premodule and postmodule survey, there was a message reminding students that by submitting the survey, they would not be able to withdraw from the study because their answers were anonymous. The study received approval from the Rutgers Institutional Review Board in August 2017.

The aim of the survey was to evaluate whether students gained nutrition knowledge from the survey and if a self-study module was an effective modality to teach nutrition. All RRWJMS students received the same standard nutrition education provided by RRWJMS during their first and second years of medical school, which did not include education on malnutrition in the hospital setting or nutrition interventions available in the hospital. The survey consisted of 10 multiple-choice questions to be completed in less than 5 minutes. The premodule survey questions aimed to determine if students had exposure to patients requiring nutrition intervention, their understanding of nutrition recommendations, their exposure to additional training in nutrition education outside of the standard first- and second-year courses at RRWJMS. The survey also had a set of five questions (questions 7-11 of Appendix B) on students' self-assessment of nutrition knowledge. Developed to assess students' knowledge of nutrition, the set of five questions was based on the information provided in the module. The questions aimed to determine if students knew objective facts about nutrition assessment that qualified a patient as malnourished and the interventions available to provide nutrition support to patients in the hospital. The questions on the postmodule survey aimed to determine if the students had completed the education module, if they were exposed to other nutrition education during the rotation, and the utility of the module. The postmodule survey also included the same set of five questions (questions 6-10 of Appendix D) on students' self-assessment of nutrition knowledge as the premodule survey. The self-assessment questions were scored by giving 0 points if students did not know the answer, 1 point if they could give a partial answer, and 2 points if they could give the full answer, for a maximum of 10 points.

Results

A total of 109 out of 255 students (43%) responded to the premodule nutrition assessment survey. Of these students, 82% reported no formal nutrition education outside of their 2 years of preclinical courses. Yet 64% had encountered cases that required nutrition intervention, and 51% did not understand the nutrition interventions recommended.

A total of 32 students reviewed the module and completed the postmodule nutrition assessment survey for a 13% response rate. Of these students, 29 reported no formal nutrition education during their clerkship. The three students who reported formal nutrition training during their rotation also reported learning new information from the module. Overall, 68% of students (21) reported learning new information that was relevant to their clerkship, and 72% reported that the module helped them feel more comfortable with cases that required nutrition intervention.

The mean nutrition knowledge sum score of the five-question self-assessment of the 109 students who took the premodule survey was a 2.4 ($SD = 1.5$) out of 10 maximal points. The mean nutrition knowledge sum score of the 32 students who took the postmodule survey was 4.5 out of 10. The independent *t* sample score was 22.7 ($p < .0001$), which indicated that the module was associated with a significant improvement in medical students' perceived self-assessment of nutrition knowledge provided in the module. Furthermore, students provided written and verbal feedback on the usefulness of the module, including the following statements:

- "This module had helpful information."
- "Before the module I did not know what was meant by nutrition support."
- "I did not know what [total parenteral nutrition] was."

Copyright © 2020 Dutra et al. This is an open-access publication distributed under the terms of the Creative Commons Attribution license.

- “The module helped me understand the different ways to provide tube feeds.”
- “I thought albumin was how we determined nutritional status.”
- “The module should be a requirement.”

Although there was no standardized education program for medical students on the clinical nutrition intervention for hospitalized patients, 64% of our students reported having encountered clinical cases requiring nutrition intervention, and 68% learned new information from the module that was applicable to their clerkship.

Discussion

In accordance with prior reports, we found that students benefited from nutrition education yet had limited exposure to nutrition education that was applicable clinically.[24-26] Our self-study module proved to be associated with a significant increase in the perceived knowledge of clinically applicable nutrition interventions in the hospital setting. While other resources—such as those from Chandra and colleagues; Elder, Perone, Branski, and Brown; and Ray and colleagues—are available for nutrition education for the inpatient setting, they are designed to be in-class learning activities that require preparation time and lecture time from staff.[37-39] Our module differs from these resources in that it provides nutrition education for the inpatient setting and has been designed to be a self-study tool requiring less time commitment from staff and students.

Medical students’ alternating clinical clerkships make it challenging to reliably have experts such as registered dietitians provide nutrition lectures at each clerkship where students are likely to encounter cases requiring nutrition interventions in the hospital setting. Furthermore, while health care systems have dietitians on staff, their role in education varies, and they are often unrecognized.[23] Given that program directors report ease of implementation and resources to be major barriers to nutrition education, our approach may prove to be an efficient and inexpensive method of delivering clinically relevant information.[33] An online training using our module as a tool could be implemented directly as part of required homework assignments that do not need to be done during a formal in-class session, or the module could be used as a tool by educators including trained staff in nutrition such as registered dietitians. With this approach, the information could be delivered to all students at a point during their clerkship time when they are most likely to benefit without requiring a lecturer to present the information in person. This would allow programs to overcome the barriers of limited faculty while giving all students an opportunity to be exposed to nutrition education in the inpatient setting via a method that fits into their busy clinical years. Students could also use the module as a quick reference tool during their clerkship. In addition, by increasing their understanding of nutrition assessment and available interventions, students could learn to appreciate the role of dietitians in a multidisciplinary team approach to patient care. This would also meet the LCME’s recommendations for the accreditation of medical education programs leading to a medical degree that emphasize interprofessional skills for medical students.[36]

Limitations

This online module was feasible and easily accessible to students. However, the major limitation of the project was the response rate of students who completed the module and postmodule survey. We believe that this was in part due to the target population, the timing of the module, and the fact that it was a volunteer activity. The module was sent primarily to third-year medical students who had to volunteer to participate out of their own interest, with no performance incentives. During the third year of medical school at RRWJMS, students were tasked with assessing patients clinically in the hospital for the first time as well as required to take multiple National Board of Medical Examiners subject exams. Therefore, we suspect that students had limited time to volunteer for additional work outside of the requirements for their rotations and exams. Moreover, the postmodule survey was sent at the end of the rotations, nearing the time of exams, which may have limited the number of students who saw or read the email and considered the completion of the survey a priority. Additionally, the self-assessment questions measured perceived knowledge instead of actual gained knowledge from the module. Another limitation to our results may be related to the time difference between when students reviewed the module and when they took the postmodule survey as it may have spanned 8 weeks. However, this could reflect information retention and therefore apply to the clinical reality that information must be consistently reviewed and utilized for retention. Furthermore, we did not ask what type of additional formal nutrition training students received to determine if they had been given education specific to nutrition support in the hospital setting. However, even if the three students who reported receiving additional training had received education specific to malnutrition and nutrition support, they reported learning new information that was helpful from the module.

Copyright © 2020 Dutra et al. This is an open-access publication distributed under the terms of the Creative Commons Attribution license.

Solutions and Future Research
Given students' verbal and written reports and assessment of valued information from the module but limited self-study time during clerkships with subject exams, we propose the following methodologies for future administration of this module:

1. Educators can consider targeting only students in clerkships who do not have associated exams and are more advanced, such as subinternships in medicine or surgery and critical care, to take advantage of students with more free time for independent study while in clerkships where they are likely to encounter patients who require nutrition interventions.
2. The postmodule survey could be submitted during the halfway point of clerkships to assess the effectiveness of the module while not coinciding with exams.
3. Alternatively, the module could be a required activity administered as part of built-in didactic time during class or home assignment, which would allow all students to complete the module without taking away from their own free study time.
4. We propose a 20-minute video lecture of the module to be delivered alongside a premodule and postmodule survey during a designated didactic session of one of the clerkships. We suspect that if the module is given during a designated didactic time as an online read-only module or video module, educators could improve overall nutrition education that is clinically applicable in the inpatient setting. However, this method needs to be assessed for its effectiveness.
5. We suggest adding a question to the survey asking at what time of the year students are taking the survey to determine if there is a temporal correlation between clinical year exposure to other nutrition education that students may receive during clerkships.
6. Lastly, to improve the assessment of the utility of the module, we recommend using a code to pair the data for the premodule and postmodule surveys and using the answer sheet (Appendix E) to grade written responses in order to assess students' actual knowledge, instead of perceived knowledge, gained from reviewing the module.

We conclude that, similar to what has been found by prior studies, medical students feel inadequately prepared to handle nutrition-related interventions clinically. We believe this module provides essential information in a succinct format that students can review in a short time frame and use as a reference tool during their clerkships and beyond. However, while an independent self-study module is easily accessible to medical students and overcomes barriers related to cost and faculty availability to teach nutrition, we must determine the best time to administer such a module and make it a required activity instead of a voluntary one.

Appendices

A. Instructions.docx

B. Premodule Nutrition Evaluation Survey.docx

C. Nutrition Education Module.pptx

D. Postmodule Nutrition Evaluation Survey.docx

E. Answer Key.docx

All appendices are peer reviewed as integral parts of the Original Publication.

Barbara Dutra, MD, RD: Resident, Department of Internal Medicine, University of Texas Health Science Center at Houston

Matthew Lissauer, MD: Associate Professor of Surgery, Department of Surgery, Rutgers Robert Wood Johnson Medical School

Hanin Rashid, PhD, EdM: Associate Director, Office for Advancing Learning, Teaching, and Assessment, Rutgers Robert Wood Johnson Medical School; Assistant Professor, Department of Psychiatry, Rutgers Robert Wood Johnson Medical School

Disclosures
None to report.

Funding/Support
None to report.

Prior Presentations
Dutra B, Lissauer M, Rashid H. Nutritional education on the wards: a self-directed module for improving medical student knowledge of nutrition assessment and interventions. Poster presented at: AAMC Northeast Group on Educational Affairs Annual Conference; April 4-6, 2019; Philadelphia, PA.

Ethical Approval
The Rutgers University Institutional Review Board approved this study.

References

1. Naber TH, Schermer T, deBree A, et al. Prevalence of malnutrition in nonsurgical hospitalized patients and its association with disease complications. *Am J Clin Nutr*. 1997;66(5):1232-1239. https://doi.org/10.1093/ajcn/66.5.1232

Copyright © 2020 Dutra et al. This is an open-access publication distributed under the terms of the Creative Commons Attribution license.

2. Robinson MK, Trujillo EB, Mogensen KM, Rounds J, McManus K, Jacobs DO. Improving nutritional screening of hospitalized patients: the role of prealbumin. *JPEN J Parenter Enteral Nutr*. 2003;27(6):389-395. https://doi.org/10.1177/0148607103027006389
3. Norman K, Pichard C, Lochs H, Pirlich M. Prognostic impact of disease-related malnutrition. *Clin Nutr*. 2008;27(1):5-15. https://doi.org/10.1016/j.clnu.2007.10.007
4. Lim SL, Ong KCB, Chan YH, Loke WC, Ferguson M, Daniels L. Malnutrition and its impact on cost of hospitalization, length of stay, readmission and 3-year mortality. *Clin Nutr*. 2012;31(3): 345-350. https://doi.org/10.1016/j.clnu.2011.11.001
5. US Burden of Disease Collaborators. The state of US health, 1990-2010: burden of diseases, injuries, and risk factors. *JAMA*. 2013;310(6):591-606. https://doi.org/10.1001/jama.2013.13805
6. Jensen GL, Mirtallo J, Compher C, et al. Adult starvation and disease-related malnutrition: a proposal for etiology-based diagnosis in the clinical practice setting from the International Consensus Guideline Committee. *JPEN J Parenter Enteral Nutr*. 2010;34(2):156-159. https://doi.org/10.1177/0148607110361910
7. White JV, Guenter P, Jensen G, Malone A, Schofield M; Academy Malnutrition Work Group; A.S.P.E.N. Malnutrition Task Force; A.S.P.E.N. Board of Directors. Consensus statement: Academy of Nutrition and Dietetics and American Society for Parenteral and Enteral Nutrition: characteristics recommended for the identification and documentation of adult malnutrition (undernutrition). *JPEN J Parenter Enteral Nutr*. 2012;36(3): 275-283. https://doi.org/10.1177/0148607112440285
8. Barker LA, Gout BS, Crowe TC. Hospital malnutrition: prevalence, identification and impact on patients and the healthcare system. *Int J Environ Res Public Health*. 2011;8(2):514-527. https://doi.org/10.3390/ijerph8020514
9. Scrimshaw NS, SanGiovanni JP. Synergism of nutrition, infection, and immunity: an overview. *Am J Clin Nutr*. 1997;66(2):464S-477S. https://doi.org/10.1093/ajcn/66.2.464S
10. Fry DE, Pine M, Jones BL, Meimban RJ. Patient characteristics and the occurrence of never events. *Arch Surg*. 2010;145(2): 148-151. https://doi.org/10.1001/archsurg.2009.277
11. Schneider SM, Veyres P, Pivot X, et al. Malnutrition is an independent factor associated with nosocomial infections. *Br J Nutr*. 2004;92(1):105-111. https://doi.org/10.1079/BJN20041152
12. Demling RH. Nutrition, anabolism, and the wound healing process: an overview. *Eplasty*. 2009;9:e9.
13. Sullivan DH, Bopp MM, Roberson PK. Protein-energy undernutrition and life-threatening complications among the hospitalized elderly. *J Gen Intern Med*. 2002;17(12):923-932. https://doi.org/10.1046/j.1525-1497.2002.10930.x
14. Corkins MR, Guenter P, DiMaria-Ghalili RA, et al.; American Society for Parenteral and Enteral Nutrition. Malnutrition diagnoses in hospitalized patients: United States, 2010. *JPEN J Parenter Enteral Nutr*. 2014;38(2):186-195. https://doi.org/10.1177/0148607113512154
15. Correia MITD, Waitzberg DL. The impact of malnutrition on morbidity, mortality, length of hospital stay and costs evaluated through a multivariate model analysis. *Clin Nutr*. 2003;22(3): 235-239. https://doi.org/10.1016/S0261-5614(02)00215-7
16. Sriram K, Sulo S, VanDerBosch G, et al. A comprehensive nutrition-focused quality improvement program reduces 30-day readmissions and length of stay in hospitalized patients. *JPEN J Parenter Enteral Nutr*. 2017;41(3):384-391. https://doi.org/10.1177/0148607116681468
17. Philipson TJ, Snider JT, Lakdawalla DN, Stryckman B, Goldman DP. Impact of oral nutritional supplementation on hospital outcomes. *Am J Manag Care*. 2013;19(2):121-128.
18. Meehan A, Loose C, Bell J, Partridge J, Nelson J, Goates S. Health system quality improvement: impact of prompt nutrition care on patient outcomes and health care costs. *J Nurs Care Qual*. 2016;31(3):217-223. https://doi.org/10.1097/NCQ.0000000000000177
19. Snider JT, Jena AB, Linthicum MT, et al. Effect of hospital use of oral nutritional supplementation on length of stay, hospital cost, and 30-day readmissions among Medicare patients with COPD. *Chest*. 2015;147(6):1477-1484. https://doi.org/10.1378/chest.14-1368
20. Somanchi M, Tao X, Mullin GE. The facilitated early enteral and dietary management effectiveness trial in hospitalized patients with malnutrition. *JPEN J Parenter Enteral Nutr*. 2011;35(2): 209-216. https://doi.org/10.1177/0148607110392234
21. Marik PE, Zaloga GP. Early enteral nutrition in acutely ill patients: a systemic review. *Crit Care Med*. 2001;29(12):2264-2270. https://doi.org/10.1097/00003246-200112000-00005
22. Devries S, Dalen JE, Eisenberg DM, et al. A deficiency of nutrition education in medical training. *Am J Med*. 2014;127(9):804-806. https://doi.org/10.1016/j.amjmed.2014.04.003
23. Kris-Etherton PM, Akabas SR, Bales CW, et al. The need to advance nutrition education in the training of health care professionals and recommended research to evaluate implementation and effectiveness. *Am J Clin Nutr*. 2014;99(5): 1153S-1166S. https://doi.org/10.3945/ajcn.113.073502
24. Frantz DJ, Munroe C, McClave SA, Martindale R. Current perception of nutrition education in U.S. medical schools. *Curr Gastroenterol Rep*. 2011;13(4):376-379. https://doi.org/10.1007/s11894-011-0202-z
25. Adams KM, Kohlmeier M, Zeisel SH. Nutrition education in U.S. medical schools: latest update of a national survey. *Acad Med*. 2010;85(9):1537-1542. https://doi.org/10.1097/ACM.0b013e3181eab71b
26. Mogre V, Stevens FCJ, Aryee PA, Amalba A, Scherpbier AJJA. Why nutrition education is inadequate in the medical curriculum: a qualitative study of students' perspectives on barriers and

Copyright © 2020 Dutra et al. This is an open-access publication distributed under the terms of the Creative Commons Attribution license.

strategies. *BMC Med Educ*. 2018;18:26. https://doi.org/10.1186/s12909-018-1130-5

27. National Research Council. *Nutrition Education in U.S. Medical Schools*. National Academies Press; 1985.
28. NAA Collaborative Group. Translation of nutritional sciences into medical education: the Nutrition Academic Award Program. *Am J Clin Nutr*. 2001;74(2):164-170. https://doi.org/10.1093/ajcn/74.2.164
29. Nutrition education for health professionals. National Heart, Lung, and Blood Institute. Accessed February 2017. https://www.nhlbi.nih.gov/events/2012/nutrition-education-health-professionals
30. Lindell KC, Adams KM, Kohlmeier M, Zeisel SH. The evolution of Nutrition in Medicine, a computer-assisted nutrition curriculum. *Am J Clin Nutr*. 2006;83(4):956S-962S. https://doi.org/10.1093/ajcn/83.4.956S
31. Nutrition in Medicine: a comprehensive online medical nutrition curriculum for training current and future healthcare professionals. *Nutrition in Medicine. Published* 2014. Accessed January 20, 2017. http://www.nutritioninmedicine.org/
32. Curriculum Committee of the Nutrition Academic Award Program. *Nutrition Curriculum Guide for Training Physicians: Practice Behavior Skills and Attitudes Across the Curriculum*. National Heart, Lung, and Blood Institute; 2002.
33. Daley BJ, Cherry-Bukowiec J, Van Way CW, et al.; A.S.P.E.N. Task Force on Postgraduate Medical Education. Current status of nutrition training in graduate medical education from a survey of residency program directors: a formal nutrition education course is necessary. *JPEN J Parenter Enteral Nutr*. 2016;40(1):95-99. https://doi.org/10.1177/0148607115571155
34. Hark LA, Deen DD, Morrison G. Learner-directed nutrition content for medical schools to meet LCME standards. *J Biomed Educ*. 2015;46935. https://doi.org/10.1155/2015/469351
35. Kushner RF, Van Horn L, Rock CL, et al. Nutrition education in medical school: a time of opportunity. *Am J Clin Nutr*. 2014;99(5):1167S-1173S. https://doi.org/10.3945/ajcn.113.073510
36. Liaison Committee on Medical Education. *Functions and Structure of a Medical School: Standards for Accreditation of Medical Education Programs Leading to the MD Degree*. Liaison Committee on Medical Education; 2018.
37. Chandra K, Lipay C, Lindsay A, et al. Introduction to nutrition care in the hospital setting. *MedEdPORTAL*. 2015;11:10213. https://doi.org/10.15766/mep_2374-8265.10213
38. Elder J, Perone J, Branski L, Brown K. Nutrition in critical care: a modified team-based learning exercise. *MedEdPORTAL*. 2016; 12:10386. https://doi.org/10.15766/mep_2374-8265.10386
39. Ray S, Udumyan R, Rajput-Ray M, et al. Evaluation of a novel nutrition education intervention for medical students from across England. *BMJ Open*. 2012;2(1):e000417. https://doi.org/10.1136/bmjopen-2011-000417

Received: October 6, 2019
Accepted: March 9, 2020
Published: October 16, 2020

Copyright © 2020 Dutra et al. This is an open-access publication distributed under the terms of the Creative Commons Attribution license.
